# Transient receptor potential (TRP) channels as drug targets for diseases of the digestive system

**DOI:** 10.1016/j.pharmthera.2011.03.006

**Published:** 2011-07

**Authors:** Peter Holzer

**Affiliations:** Research Unit of Translational Neurogastroenterology, Institute of Experimental and Clinical Pharmacology, Medical University of Graz, Universitätsplatz 4, A-8010 Graz, Austria

**Keywords:** Chemesthesis, Chemosensation, Gastrointestinal cancer, Gastrointestinal motility, Hypersensitivity, Hyperalgesia, Inflammation, Inflammatory bowel disease, Mechanosensation, Pain, Taste, Transducers, TRPA1, TRPC4, TRPC6, TRPM5, TRPM6, TRPV1, TRPV4, TRPV6, AITC, allyl isothiocyanate, CCK, cholecystokinin, CGRP, calcitonin gene-related peptide, DRG, dorsal root ganglion, DSS, dextran sulfate sodium, GI, gastrointestinal, GPCR, G protein-coupled receptor, 5-HT, 5-hydroxytryptamine, ICC, interstitial cell of Cajal, mRNA, messenger ribonucleic acid, PAR, protease-activated receptor, PKD, polycystic kidney disease, RNA, ribonucleic acid, siRNA, small interfering ribonucleic acid, TNBS, trinitrobenzene sulfonic acid, TRP, transient receptor potential, TRPA, transient receptor potential ankyrin, TRPC, transient receptor potential canonical (or classical), TRPM, transient receptor potential melastatin, TRPP, transient receptor potential polycystin, TRPV, transient receptor potential vanilloid

## Abstract

Approximately 20 of the 30 mammalian transient receptor potential (TRP) channel subunits are expressed by specific neurons and cells within the alimentary canal. They subserve important roles in taste, chemesthesis, mechanosensation, pain and hyperalgesia and contribute to the regulation of gastrointestinal motility, absorptive and secretory processes, blood flow, and mucosal homeostasis. In a cellular perspective, TRP channels operate either as primary detectors of chemical and physical stimuli, as secondary transducers of ionotropic or metabotropic receptors, or as ion transport channels. The polymodal sensory function of TRPA1, TRPM5, TRPM8, TRPP2, TRPV1, TRPV3 and TRPV4 enables the digestive system to survey its physical and chemical environment, which is relevant to all processes of digestion. TRPV5 and TRPV6 as well as TRPM6 and TRPM7 contribute to the absorption of Ca^2+^ and Mg^2+^, respectively. TRPM7 participates in intestinal pacemaker activity, and TRPC4 transduces muscarinic acetylcholine receptor activation to smooth muscle contraction. Changes in TRP channel expression or function are associated with a variety of diseases/disorders of the digestive system, notably gastro-esophageal reflux disease, inflammatory bowel disease, pain and hyperalgesia in heartburn, functional dyspepsia and irritable bowel syndrome, cholera, hypomagnesemia with secondary hypocalcemia, infantile hypertrophic pyloric stenosis, esophageal, gastrointestinal and pancreatic cancer, and polycystic liver disease. These implications identify TRP channels as promising drug targets for the management of a number of gastrointestinal pathologies. As a result, major efforts are put into the development of selective TRP channel agonists and antagonists and the assessment of their therapeutic potential.

## 1 Introduction

The selection of useful food and its effective digestion are essential for survival. To accomplish this goal, the digestive tract possesses a large array of molecular sensors and transducers to make the right choice and guide the process of digestion by appropriate feed-forward and feed-back mechanisms. The physiological task of the alimentary canal is highly challenging because, depending on the nature of ingested food, the gut needs to carry out programs with opposite aims: digestion of food and absorption of nutrients and water, on the one hand, and the elimination of toxins, antigens, pathogens and indigestible waste, on the other hand. Research in the past decade has revealed an elaborate system of molecular sensors serving taste, analyzing the chemical composition of food, monitoring the motor and secretory activity of the gastrointestinal (GI) tract, and detecting adverse conditions in the lumen and wall of the alimentary canal. Transient receptor potential (TRP) channels, so named after the role they play in *Drosophila* phototransduction, take a prominent position among these sensors and transducers in the digestive system. A number of their implications in GI physiology and pathology were discovered subsequently to the analysis of the mechanism of action of pungent spices (Bandell et al., 2007; Holzer, 2008a).

The search for and the intake of nutrients are governed by the appearance, smell and taste of food. There is anthropological evidence that the adoption of cooking food has been a key feature in human evolution (Wrangham & Conklin-Brittain, 2003; Wobber et al., 2008). Along with cooking, mankind also learned that the taste of food can be heightened by seasoning, and food prepared with the right mixture of spices is one of the major human pleasures. The chemicals responsible for the gustatory and olfactory pleasures of spices are secondary metabolites of plants (Max, 1992; Cromer & McIntyre, 2008; Vriens et al., 2008). Recognition of the chemical qualities of spices must have driven the co-evolution of TRP channels as appropriate sensors in the animal kingdom. The functional implications of TRP channels are, in the first place, congruent with the strategy of plants to discourage predators by the unusual sensory quality of spices. However, by perversion of this principle, humans have learned to enjoy low doses of those deterrent chemicals (Max, 1992). This argument is supported by the fact that capsaicin is pungent for mammals, but not birds which are supposed to help distributing the seeds of red pepper, given that the avian orthologue of the TRP vanilloid-1 channel (TRPV1) lacks the binding site for capsaicin (Jordt & Julius, 2002).

The implication of TRP channels in the sensation of deterrent chemicals was first heralded when, in 1997, TRPV1 (then named the vanilloid receptor-1) was identified as the sensor for capsaicin at the genetic and functional level (Caterina et al., 1997). Responsible for the piquancy of red pepper (*Capsicum* spp.), the vanilloid capsaicin in its pure form is one of the most painful chemicals we know, yet is also widely used for food seasoning. TRPV1 was soon joined by other TRP channels with unique transduction properties relevant to chemo-, thermo- and mechanosensation as well as sweet, bitter, sour, salt and umami taste sensation (Zhang et al., 2003; DeSimone & Lyall, 2006; Huang et al., 2006; Bandell et al., 2007; Kaske et al., 2007; Montell & Caterina, 2007; Vriens et al., 2008; Ishimaru & Matsunami, 2009; Wu et al., 2010). In addition, these molecular sensors can detect specific chemical entities including unpleasant and/or painful toxins, whereby they subserve chemesthesis, the chemical sense distinct from taste and smell (Bandell et al., 2007).

Currently, some 30 different TRP subunit genes have been identified in mammals, these subunits being grouped in 6 families (Wu et al., 2010). Members of 5 subunit families, specifically of the vanilloid TRP (TRPV), melastatin TRP (TRPM), ankyrin TRP (TRPA), polycystin TRP (TRPP) and canonical or classical TRP (TRPC) family, are relevant to spice sensing, chemo-, thermo- and/or mechanosensation (Table 1). The TRP channel subunits are made of 6 transmembrane domains with a pore between transmembrane domains 5 and 6 (Clapham et al., 2005; Wu et al., 2010). Functional TRP channels, thought to be composed as tetramers of four subunits, are opened or closed by conformational changes in the channel protein (Dhaka et al., 2006; Bandell et al., 2007). TRP channels are only weakly sensitive to depolarization but open in response to changes in temperature, binding of ligands or other alterations of the channel protein (Clapham et al., 2005; Matta & Ahern, 2007; Nilius et al., 2007; Wu et al., 2010). The ion selectivity differs markedly among the family of TRP channels, most of them being non-selective cation channels.

**Fig. 1:**
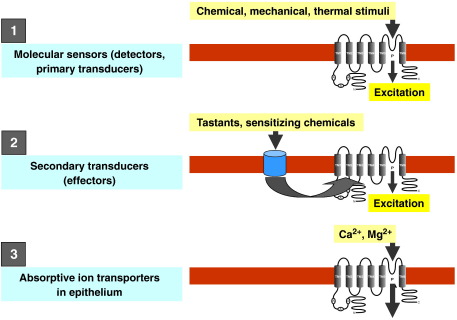
Diagram portraying 3 different molecular roles of TRP channels in the digestive system: (**1**) TRP channels as molecular sensors (detectors or primary transducers) of chemical and physical stimuli, (**2**) TRP channels as secondary transducers (downstream transducers or effectors) of cell activation induced by G protein-coupled receptors or ion channel receptors, and (**3**) TRP channels as Ca^2+^ or Mg^2+^ transport channels.

**Fig. 2:**
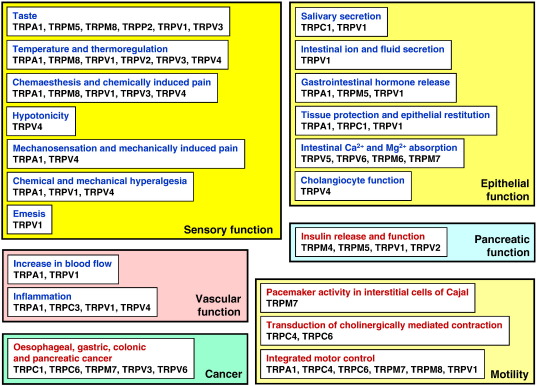
Overview of the functional implications of TRP channels in the digestive system.

**Fig. 3:**
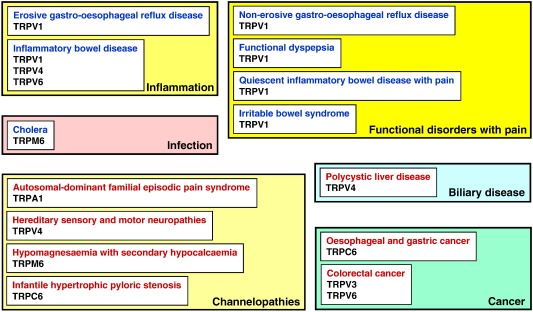
Overview of the associations of TRP channels with diseases and disorders of the digestive system.

**Fig. 4:**
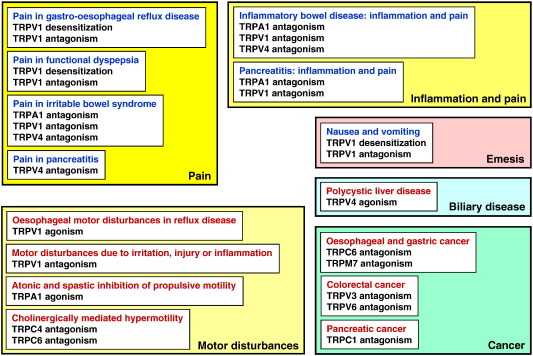
Overview of the therapeutic potential of TRP channel ligands in diseases and disorders of the digestive system.

The objective of this article is to review the occurrence and function of TRP channels in the digestive tract, with a particular emphasis on their roles in health and disease. To keep the wealth of the available information in perspective, overview panels guide the reader to keep track of the functional implications of TRP channels in the alimentary canal (Figs. 1 and 2) and their associations with disorders of the digestive system (Fig. 3). Alterations in their expression and function in disease have raised enormous interest in TRP channels being attractive targets for new therapeutic strategies in gastroenterology. Accordingly, several TRP channel ligands have been developed, and it is a further objective of this article to summarize and discuss the pharmacological opportunities which TRP channel ligands offer to gastroenterology (Fig. 4).

## 2 Expression and functional implications of TRP channels in the digestive tract

### 2.1 General considerations

Although various TRP channels, notably TRPV1, TRPV3, TRPA1, TRPM5 and TRPM8, enable humans to enjoy seasoned food, they subserve a much wider spectrum of functions in the alimentary canal. TRP channels survey the environment of the gut for a large variety of chemicals and toxins that either come in with the food, are formed in the GI tissue, are constituents of the digestive juice, are produced by the GI microbiome, or are generated by tissue injury and inflammation (Table 1). In addition, some TRP channels are involved in mechanosensation or can sense a specific range of temperatures. Apart from their sensory surveillance function, TRP channels in the alimentary canal are relevant to the control of membrane potential and excitability of neurons, epithelial cells, muscle cells and interstitial cells of Cajal (ICCs), play a role in Ca^2+^ and Mg^2+^ absorption, govern blood flow, pacemaker activity, motor activity, secretory processes and mucosal homeostasis, and impact on the development of GI cancer.

In reviewing the functional implications of TRP channels in the alimentary canal, 3 distinct roles come to light (Fig. 1). (1) TRP channels operate as molecular sensors (detectors or primary transducers) of chemical and physical stimuli; (2) TRP channels play a role as downstream or secondary transducers (or effectors) of cell activation mediated by G protein-coupled receptors (GPCRs) or ion channel receptors; and (3) TRP channels function as ion transport channels responsible, e.g., for Ca^2+^ and Mg^2+^ homeostasis. Within primary afferent neurons, translation of signals detected by TRP channel detectors into effector responses is carried by two distinct mechanisms: (1) by the local release of neuropeptides from the peripheral fibers of TRP-expressing afferent neurons, which causes changes in local tissue function (Holzer, 1988; Maggi & Meli, 1988); and (2) by transmission to the central nervous system resulting in autonomic reflex responses and sensation.

### 2.2 TRPV1 channels

#### 2.2.1 Occurrence of TRPV1 in the alimentary canal

The existence of TRPV1, the specific sensor for capsaicin, has been envisaged more than 30 years ago, given that most pharmacological effects of capsaicin have been attributed to a specific action on *capsaicin-sensitive* primary afferent neurons (Jancsó et al., 1987; Holzer, 1991; Szallasi & Blumberg, 1999; Szolcsányi, 2004). The role of *capsaicin-sensitive* afferent neurons and, by indirect inference, the distribution of *capsaicin receptors* have been traced with two experimental paradigms: stimulation of afferent neurons by acute application of capsaicin, and pretreatment of animals or tissues with high doses of capsaicin (“capsaicin desensitization”) to defunctionalize afferent neurons via excess influx of Ca^2+^ following stimulation of TRPV1. *Capsaicin-sensitive* nerve cells may even degenerate if, e.g., a high dose of capsaicin is administered to newborn rats (Jancsó et al., 1977) or micromolar concentrations of capsaicin are added to dorsal root ganglion (DRG) cultures (Wood et al., 1988). Since the whole sensory neuron is defunctionalized by this approach, referral to a *capsaicin-sensitive* structure or function in its historical connotation does not necessarily point to an implication of TRPV1. In addition, capsaicin at higher concentrations can act on ion channels unrelated to TRPV1 and on cellular structures lacking TRPV1 (Holzer, 1991) such as ICCs (Choi et al., 2010). Treatment of adult rats with a neurotoxic dose of capsaicin reduces the number of nodose ganglion neurons that express TRPV1 and project to the stomach as observed 10 days post-treatment. By 67 days post-treatment, much of the vagal afferent innervation of the stomach is restored, mainly because of sprouting of capsaicin-insensitive nerve fibers (Ryu et al., 2010).

In the alimentary canal, TRPV1 occurs in extrinsic sensory neurons, intrinsic enteric neurons as well as epithelial and endocrine cells. The major cellular sources of TRPV1 in the rat, guinea-pig and murine alimentary canal are spinal and vagal primary afferent neurons (Patterson et al., 2003; Ward et al., 2003; Holzer, 2004; Horie et al., 2004; Kadowaki et al., 2004; Robinson et al., 2004; Schicho et al., 2004; Banerjee et al., 2007; Ryu et al., 2010; Zhao et al., 2010). Both in the DRG and nodose ganglion cells, TRPV1 is restricted to small and medium-sized somata that are known to give rise to unmyelinated (C-) and thinly myelinated (Aδ-) fibers (Caterina et al., 1997; Guo et al., 1999; Michael & Priestley, 1999; Banerjee et al., 2007; Tan et al., 2008, 2009). There are distinct regional differences in the relative proportion of sensory neurons that stain positive for TRPV1. Thus, TRPV1-immunoreactive fibers are considerably more prevalent in visceral than in somatic afferent neurons (Robinson et al., 2004; Brierley et al., 2005; Hwang et al., 2005; Christianson et al., 2006a). In fact, the majority of nodose ganglion neurons projecting to the rodent stomach and of DRG neurons projecting to the rodent gut express TRPV1 (Robinson et al., 2004; Schicho et al., 2004; Zhang et al., 2004; Brierley et al., 2005; Hwang et al., 2005; Christianson et al., 2006a, 2006b; Tan et al., 2008; Zhong et al., 2008), while only 32% of the vagal afferent neurons supplying the murine jejunum contain TRPV1 (Tan et al., 2009).

Within the rodent and human alimentary canal, TRPV1-positive nerve fibers occur in the musculature, enteric nerve plexuses, arterioles and mucosa (Facer et al., 2001; Yiangou et al., 2001; Chan et al., 2003; Patterson et al., 2003; Ward et al., 2003; Holzer, 2004; Horie et al., 2004; Kadowaki et al., 2004; Matthews et al., 2004; Robinson et al., 2004; Schicho et al., 2004; Zhang et al., 2004; Faussone-Pellegrini et al., 2005; Bhat & Bielefeldt, 2006; Akbar et al., 2008; Matsumoto et al., 2009, 2011). In addition, TRPV1 is present in nerve fibers supplying the taste papillae of the tongue (Caterina et al., 1997; Tominaga et al., 1998; Ishida et al., 2002; DeSimone & Lyall, 2006).

There are regional and species differences in the chemical coding of primary afferent neurons expressing TRPV1. Thus, calcitonin gene-related peptide (CGRP), substance P, somatostatin and other neuropeptides are messenger molecules characteristic of *capsaicin-sensitive* DRG neurons (Green & Dockray, 1988; Holzer, 1991; Sternini, 1992; Szallasi & Blumberg, 1999; Hwang et al., 2005; Price & Flores, 2007), while CGRP is absent from vagal afferent neurons containing TRPV1 (Tan et al., 2009). DRG neurons can be largely differentiated by their binding of isolectin B_4_ and their responsiveness to different neurotrophins (Guo et al., 1999; Michael & Priestley, 1999; Liu et al., 2004; Hwang et al., 2005; Price & Flores, 2007). In adult rodents, the isolectin B_4_-negative cell population responds to nerve growth factor, while isolectin B_4_-positive cells respond to the glial cell line-derived family of neurotrophins. However, there is no clear distinction between these groups of DRG neurons in their expression of TRPV1 and the neuropeptides substance P, CGRP and somatostatin (Price & Flores, 2007). In the rat, TRPV1 is found in both populations of DRG neurons but is more prevalent in isolectin B_4_-positive cells (Guo et al., 1999; Michael & Priestley, 1999; Liu et al., 2004; Hwang et al., 2005; Banerjee et al., 2007; Price & Flores, 2007), whereas in the mouse TRPV1 is largely absent from isolectin B_4_-positive DRG cells (Zwick et al., 2002; Woodbury et al., 2004; Price & Flores, 2007). In both rat and mouse, however, TRPV1 abounds in visceral DRG neurons that bind little isolectin B_4_ but are rich in CGRP and substance P (Ward et al., 2003; Robinson et al., 2004; Schicho et al., 2004; Brierley et al., 2005; Hwang et al., 2005; Christianson et al., 2006a, 2006b; Tan et al., 2008).

The presence of TRPV1 in the enteric nervous system has been less extensively studied than that in primary afferent neurons and has remained a matter of controversy. While some authors have localized TRPV1 immunoreactivity to somata of the guinea-pig, porcine and human enteric nervous system (Nozawa et al., 2001; Poonyachoti et al., 2002; Anavi-Goffer & Coutts, 2003; Chan et al., 2003), other authors have failed to confirm this location (Patterson et al., 2003; Ward et al., 2003; Holzer, 2004; Schicho et al., 2004). Importantly, the chemical coding of TRPV1-positive DRG neurons innervating the murine gut and of TRPV1-positive nerve fibers in the murine colon and rectum is distinct from that of enteric neurons (Tan et al., 2008; Matsumoto et al., 2011). Furthermore, TRPV1 messenger ribonucleic acid (mRNA) disappears from the rat stomach following extrinsic denervation (Schicho et al., 2004). Enteric neurons that issue projections from the rat colon to the spinal cord are likewise TRPV1-negative (Suckow & Caudle, 2008). The inconsistencies in the localization of TRPV1 to enteric neurons could in part be explained by the existence of several splice variants of TRPV1 (Wang et al., 2004; Faussone-Pellegrini et al., 2005; Szallasi et al., 2007) that may substantially differ in their immunoreactivity. For instance, two splicing products of the murine TRPV1 gene have been identified in the DRGs and several tissues including the gut. One of them, TRPV1-α, is equivalent to the rat and human TRPV1, whereas the other one, TRPV1-β, encodes a dominant negative subunit of the TRPV1 channel (Wang et al., 2004).

In addition to its prominent location in neurons, TRPV1 has been localized to non-neural cells of the alimentary canal. Thus, TRPV1-like immunoreactivity is found in the human submandibular gland where it occurs in serous acinar and ductal cells, but not in mucous acinar cells (Ding et al., 2010). Furthermore, TRPV1-like immunoreactivity has been localized to gastrin and parietal cells of the stomach as well as to epithelial cells of the esophageal, gastric and small intestinal mucosa of several species including man (Nozawa et al., 2001; Kato et al., 2003; Zhang et al., 2004; Faussone-Pellegrini et al., 2005; Kechagias et al., 2005; Cheng et al., 2009; Ericson et al., 2009; Ma et al., 2010). With respect to the presence of TRPV1 in these tissues a word of caution is in place, given that TRPV1 antisera can yield false positive staining in non-neural tissues, which persists in TRPV1 knockout mice (Everaerts et al., 2009).

#### 2.2.2 Functional implications of TRPV1 in the digestive system

##### 2.2.2.1 Sensory modalities of TRPV1

TRPV1 is a sensor that is stimulated by several spices, noxious heat, and a number of endogenous stimuli, some of which are particularly relevant to the GI tract (Table 1). Among the spices acting on TRPV1, capsaicin (red pepper), piperine (black pepper), gingerol and zingerone (ginger), pungent compounds from onion and garlic, eugenol (clove) and camphor figure most prominently. TRPV1 is also activated by allyl isothiocyanate present in mustard, horseradish and wasabi (Everaerts et al., 2011), resiniferatoxin, a toxin of tropical *Euphorbia* plants (Szallasi & Blumberg, 1999), and “vanillotoxins” present in the venoms of the Trinidad Chevron tarantula and Earth Tiger tarantula (Cromer & McIntyre, 2008; Bohlen et al., 2010). A taste variant of TRPV1 is thought to be involved in nonspecific salt taste perception (DeSimone & Lyall, 2006). Furthermore, TRPV1 contributes to the aversive bitter aftertaste sensation evoked by high concentrations of artificial sweeteners such as saccharin and acesulfame-K and to the metallic taste sensation evoked by high concentrations of CuSO_4_, ZnSO_4_ and FeSO_4_ (Riera et al., 2007). The burning sensation evoked by ethanol in the mouth and throat is also most likely mediated by TRPV1 (Trevisani et al., 2002). The endogenous stimuli that TRPV1 responds to include acid (pH < 6), ammonia (pH > 8), anandamide and other arachidonic acid-derived metabolites such as N-arachidonoyl-dopamine, N-oleoyldopamine, oleoylethanolamide, 12-(S)-hydroperoxy eicosatetraenoic acid, 15-(S)-hydroperoxy eicosatetraenoic acid and leukotriene B_4_ (Tominaga et al., 1998; Caterina & Julius, 2001; Holzer, 2007; Szallasi et al., 2007; Dhaka et al., 2009).

##### 2.2.2.2 Role of TRPV1 in abdominal pain and hypersensitivity

Several lines of evidence indicate that TRPV1 is involved in pain and hyperalgesia throughout the alimentary canal. In addition, TRPV1-expressing vagal afferents in the esophagus may contribute to the cough evoked by gastro-esophageal reflux of gastric contents (Kollarik & Brozmanova, 2009). In keeping with its nociceptive role, activation of TRPV1 on primary afferent neurons innervating the gut elicits pain in humans and pain-related behavior in rodents (Table 2). Intraesophageal instillation of capsaicin in human volunteers evokes symptoms of heartburn, while the sensitivity to balloon distension or acid exposure remains unchanged (Kindt et al., 2009; Chen et al., 2010). Intragastric administration of capsaicin in humans increases the sensitivity to proximal gastric distension (Lee et al., 2004), and ingestion of capsaicin capsules induces gastric sensations of pressure, heartburn and warmth (Hammer & Vogelsang, 2007). Infusion of capsaicin into the proximal small intestine of human volunteers likewise evokes sensations of pain, cramps, pressure, warmth and nausea (Schmidt et al., 2004; Hammer & Vogelsang, 2007). The capsaicin-induced sensations are most intense in the duodenum and appear to decrease along the intestine (Hammer & Vogelsang, 2007), although local capsaicin instillation in ileostomy and colostomy patients is also painful (Drewes et al., 2003; Arendt-Nielsen et al., 2008). Ingestion of capsaicin for 7 days leads to a decrease in jejunal mechanosensation and to an increase in the jejunal sensitivity to capsaicin (Hammer, 2006a), and oral chili intake for 3 days has been reported to increase rectal sensitivity to urgency (Gonlachanvit et al., 2007). In contrast, ingestion of capsaicin (0.25 mg) capsules three times per day for 4 weeks leads to desensitization of the duodenum to both acute capsaicin administration and balloon distension (Führer & Hammer, 2009).

TRPV1 can be sensitized by many proalgesic factors (Caterina & Julius, 2001; Geppetti & Trevisani, 2004; Szallasi et al., 2007; Holzer, 2008b), and there is increasing evidence that GI inflammation causes chemical and mechanical hyperalgesia at least in part by upregulation and sensitization of TRPV1 (Table 2). Neurotrophins, various inflammatory mediators as well as endocrine factors of the GI mucosa are thought to be involved in this process. Thus, the ability of 5-hydroxytryptamine (5-HT) to sensitize GI afferent neurons to heat, acid and capsaicin is absent in TRPV1 knockout mice (Sugiura et al., 2004). The effect of 5-HT to sensitize colonic DRG neurons is mediated by metabotropic 5-HT_2_ and 5-HT_4_ receptors which appear to enhance TRPV1 activity by downstream phosphorylation pathways (Sugiura et al., 2004). Vice versa, depletion of 5-HT from the colon by pretreatment of rats with p-chlorophenylalanine reduces the excitability of DRG neurons by capsaicin and elevates the threshold of distension-induced pain behavior (Qin et al., 2010). Referred hyperalgesia associated with caerulein-induced pancreatitis in mice depends on both protease-activated receptor-2 (PAR-2) and TRPV1, TRPV1 being a downstream transducer of the pronociceptive action of PAR-2 stimulation (Fukushima et al., 2010; Nishimura et al., 2010).

TRPV1 is upregulated not only in inflammation, but also in the absence of overt inflammation (Table 2) as is typical of functional GI disorders (Holzer, 2008a). This is true for patients with irritable bowel syndrome in which the increased density of TRPV1 in the rectosigmoid colon correlates with pain severity (Akbar et al., 2008). A similar correlation between pain intensity and number of mucosal TRPV1-positive nerve fibers is found in patients with quiescent inflammatory bowel disease who continue to complain of abdominal pain (Akbar et al., 2010). Non-erosive reflux disease (Bhat & Bielefeldt, 2006; Guarino et al., 2010), idiopathic rectal hypersensitivity and fecal urgency (Chan et al., 2003) are other instances of TRPV1 upregulation in the absence of appreciable inflammation. In addition, patients with functional dyspepsia (Hammer et al., 2008) and diarrhea-predominant irritable bowel syndrome (Gonlachanvit et al., 2009) exhibit hypersensitivity to the painful sensations evoked by capsaicin-containing capsules. Experimental findings have likewise shown that TRPV1 has a bearing on post-inflammatory colonic hyperalgesia in rodents, given that upregulation of TRPV1 expression and function persists long after the initial inflammatory insult has subsided (Eijkelkamp et al., 2007; Jones et al., 2007; Winston et al., 2007; Holzer, 2008b).

Although the participation of TRPV1 in chemical nociception figures most prominently, there is evidence that TRPV1 also contributes to mechanical hyperalgesia in the inflamed gut (Table 2). As shown for pelvic afferents innervating the murine colon, TRPV1 is preferentially expressed by mechanoreceptors that respond to distension with a low frequency of firing, the distension responses of these fibers being sensitized by capsaicin or acidosis (Malin et al., 2009). Pelvic afferents innervating the smooth muscle and myenteric plexus of the murine rectum are likewise sensitive to low-threshold distension and capsaicin (Spencer et al., 2008). Accordingly, capsaicin enhances the visceromotor response to colorectal distension (an indirect measure of abdominal pain), this effect being inhibited by the TRPV1 blocker SB-705,498 (van den Wijngaard et al., 2009a). Similarly, the hypersensitivity to colorectal distension, which is seen after acute stress exposure of adult rats that have been subjected to maternal separation as neonates, is reversed by TRPV1 blockade (van den Wijngaard et al., 2009b). This finding is consistent with a report that chronic exposure of adult rats to water avoidance stress for 10 days causes a significant upregulation of TRPV1 (and TRPA1) in DRG neurons supplying the colon, along with an increase in the abdominal withdrawal reflex to colorectal distension (Yu et al., 2010). Furthermore, TRPV1 blockade prevents the development of mechanical hyperalgesia that is evoked by repetitive colorectal distension in rats, but does not cause hypoalgesia (Ravnefjord et al., 2009). High-threshold splanchnic afferents innervating the mesentery and serosa of the colon fail to be sensitized in rats with dextran sulfate sodium (DSS)-induced colitis, yet the response of these fibers to mechanical probing is attenuated by a TRPV1 blocker in inflammation but not health (Phillis et al., 2009). The role of TRPV1 in mechanical pain seems to be that of a secondary transducer, because a mechanodetector role of TRPV1 has not yet been substantiated. The implication of TRPV1 in inflammatory hypersensitivity is likewise that of a secondary transducer, TRPV1 channel activity being enhanced by multiple mechanisms triggered by receptors for inflammatory mediators and neurotrophins.

##### 2.2.2.3 Role of TRPV1 in emesis

A prominent role of vagal afferent neurons supplying the upper GI tract is to cause nausea and vomiting if the ingested food is deemed potentially or actually toxic. In view of their broad spectrum of sensory modalities, distinct TRP channels are likely to play a role in these processes. There is indeed experimental evidence that activation of TRPV1 has an impact on emesis. Thus, subcutaneous or intracerebroventricular administration of capsaicin or resiniferatoxin has been reported to cause retching and vomiting in the dog, ferret and *Suncus marinus*, the house musk shrew (Shiroshita et al., 1997; Andrews et al., 2000; Rudd & Wai, 2001; Cheng et al., 2005). The proemetic action of TRPV1 activation appears to reverse quickly to an antiemetic action, given that vomiting caused by stimulation of the medial solitary nucleus in the brainstem, radiation, systemic administration of cisplatin, loperamide or apomorphine or intragastric administration of CuSO_4_ is depressed by capsaicin and resiniferatoxin (Andrews & Bhandari, 1993; Shiroshita et al., 1997; Andrews et al., 2000; Rudd & Wai, 2001; Yamakuni et al., 2002). Since the mechanism behind the dual proemetic and antiemetic action of TRPV1 agonists is not understood, the existence of a novel vanilloid receptor has been envisaged (Cheng et al., 2005). Non-pungent agonists at TRPV1 such as arvanil, olvanil, anandamide and N-arachidonoyl-dopamine fail to evoke emesis in the ferret but are able to blunt vomiting evoked by morphine-6-glucuronide, apomorphine, cisplatin and CuSO_4_ (Sharkey et al., 2007; Chu et al., 2010).

##### 2.2.2.4 Thermoregulation by abdominal TRPV1

TRPV1 is a heat sensor (Caterina et al., 1997; Tominaga et al., 1998), which explains why the sensation caused by capsaicin is described as “hot” and “burning”. Although capsaicin and related TRPV1 agonists evoke a thermoregulatory response (Szallasi & Blumberg, 1999; Masamoto et al., 2009), TRPV1 knockout mice have a normal body temperature and do not seem to have a deficit in heat sensing (Szelényi et al., 2004; Woodbury et al., 2004; Iida et al., 2005), except that heat hyperalgesia in response to inflammation (Caterina et al., 2000; Davis et al., 2000) or heat injury (Bölcskei et al., 2005) and fever in response to the bacterial pyrogen lipopolysaccharide (Iida et al., 2005) are attenuated. The maintenance of basal thermoregulation in TRPV1 knockout mice is probably due to developmental compensations, given that many TRPV1 blockers cause substantial hyperthermia in mice, rats, dogs, monkeys and humans (Gavva et al., 2007, 2008; Steiner et al., 2007; Holzer, 2008b; Garami et al., 2010). It need be inferred, therefore, that TRPV1 is tonically active to control core body temperature (Gavva et al., 2007; Montell & Caterina, 2007), lowering body temperature through suppression of autonomic cold-defense effectors (Gavva, 2008; Romanovsky et al., 2009).

The hyperthermic action of TRPV1 blockers involves cutaneous vasoconstriction and shivering-related thermogenesis, but not warmth-seeking behavior, and is inhibited by the antipyretic drug acetaminophen (Steiner et al., 2007; Gavva et al., 2008). Because the magnitude of the hyperthermic effect of the TRPV1 blocker AMG0347 is independent of the baseline temperature, it has been concluded that the rise of body temperature results from blockade of tonic TRPV1 activation by non-thermal factors (Steiner et al., 2007). Further analysis suggests that the hyperthermic response to TRPV1 antagonists depends on blockade of the proton mode of TRPV1 activation, either alone or together with the capsaicin mode of TRPV1 activation (Romanovsky et al., 2009; Garami et al., 2010). However, this issue has not yet been settled because some TRPV1 antagonists that do not block the proton mode of TRPV1 activation can elicit hyperthermia (Gavva et al., 2007) and other TRPV1 blockers that do not cause hyperthermia affect the proton mode of TRPV1 stimulation (Lehto et al., 2008; Garami et al., 2010; Voight & Kort, 2010). It thus awaits to be confirmed that hyperthermia is in fact driven by a low tissue pH, either alone or together with endovanilloid agonists (Garami et al., 2010) and that the TRPV1-mediated thermoregulatory action takes place in the abdominal cavity (Steiner et al., 2007) in which the stomach and colon have an acidic environment (Holzer, 2007). The increase in metabolic rate and thermogenesis evoked by intrajejunal administration of non-pungent capsaicin analogs (capsinoids) to mice also seems to be mediated by TRPV1 within the GI tract (Kawabata et al., 2009).

##### 2.2.2.5 Control of digestive functions by TRPV1

###### 2.2.2.5.1 Vasodilatation, tissue protection and inflammation

When TRPV1-expressing sensory nerve fibers are activated, they release peptide transmitters from their peripheral endings and in this way modify GI vascular, immune and smooth muscle functions (Holzer, 1998; Barthó et al., 2004, 2008; Holzer, 2004; Mózsik et al., 2007). Following tissue irritation or injury, some of these reactions (e.g., vasodilatation and plasma protein extravasation) contribute to the process of *neurogenic* inflammation. The TRPV1 agonist capsaicin and mustard oil (a TRPA1 and TRPV1 agonist) have been instrumental in discovering and analyzing this phenomenon (Holzer, 1988). The messengers involved in the efferent-like mode of operation include CGRP, somatostatin and the tachykinins substance P and neurokinin A (Maggi, 1995; Holzer & Maggi, 1998; Pintér et al., 2006).

Administration of capsaicin to the esophageal, gastric and intestinal mucosa increases mucosal blood flow, a response which is mimicked by exposure to excess acid (Holzer, 1998, 2004). The acid- and carbon dioxide-evoked hyperemia in the esophageal and duodenal mucosa is inhibited by the TRPV1 antagonist capsazepine, which indicates that acid activates TRPV1 on sensory nerve fibers (Akiba et al., 2006a, 2008). Through this mechanism, which also includes increases in bicarbonate and mucus secretion, TRPV1-positive sensory nerve fibers are able to protect the esophageal, gastric and intestinal mucosa from a variety of injurious insults (Holzer, 1998). Paradoxically, knockout of TRPV1 has been reported to ameliorate acid-induced injury in the esophagus and stomach (Akiba et al., 2006b; Fujino et al., 2006). Analysis of this observation in the stomach suggests that disruption of the TRPV1 gene causes a compensatory upregulation of several protective mechanisms in the gastric mucosa (Akiba et al., 2006b). These counterregulatory mechanisms may also explain why acid-induced release of CGRP from the murine stomach remains unchanged in TRPV1 knockout mice (Auer et al., 2010).

Apart from protecting the GI mucosa (Holzer, 1998; Massa et al., 2006; Martelli et al., 2007), TRPV1 activation has been found to exacerbate inflammation and injury in certain models of ileitis, colitis and pancreatitis (Table 2). In addition, capsaicin- as well as acid-evoked stimulation of epithelial TRPV1 in the feline and human esophagus has the potential to give rise to esophagitis (Cheng et al., 2009; Ma et al., 2010). In the murine gastric mucosa, ethanol-induced injury appears to involve TRPV1-mediated release of neuronal substance P and subsequent formation of reactive oxygen species (Gazzieri et al., 2007). Experimental colitis in the mouse is attenuated by TRPV1 antagonism or knockout (Table 2). Emerging evidence indicates that TRPV1 contributes to pancreatic islet inflammation associated with type I diabetes and, in addition, plays a role in insulin-dependent glucose regulation, type II diabetes, adipogenesis and obesity (Razavi et al., 2006; Gram et al., 2007; Zhang L.L. et al., 2007; Suri & Szallasi, 2008). The observation that stimulation of TRPV1, under some conditions, reduces and, under other conditions, exaggerates tissue inflammation and injury may reflect stimulus- and tissue-dependent differences in the process of neurogenic inflammation. In particular, the peptide mediators of TRPV1-positive afferent neurons cover a wide spectrum of actions: Tachykinins facilitate inflammation, while CGRP promotes vasodilatation but not necessarily inflammation, and somatostatin is capable of inhibiting inflammatory processes (Pintér et al., 2006).

###### 2.2.2.5.2 Motor activity and secretory processes

The messengers released from capsaicin-sensitive afferent nerve fibers in the gut act on enteric nervous system, GI smooth muscle and epithelium to modify motility and secretion (Weber et al., 2001; Holzer, 1998, 2002; Benkó et al., 2005; Geber et al., 2006; Schicho et al., 2006; Sibaev et al., 2006; Boudaka et al., 2007; Barthó et al., 2008; de Man et al., 2008; Matsumoto et al., 2011). Tachykinins and adenosine triphosphate stimulate motor activity, while CGRP, vasoactive intestinal polypeptide and nitric oxide account for motor inhibition caused by TRPV1 activation (Benkó et al., 2005; Barthó et al., 2008; de Man et al., 2008; Matsumoto et al., 2009). Local motor effects mediated by TRPV1 may become operative when the GI tract is disturbed by endogenous or exogenous irritants. For instance, TRPV1 and substance P are involved in the acid-evoked contraction of opossum esophageal longitudinal muscle (Paterson et al., 2007). The circular muscle of the isolated human gut is relaxed by capsaicin, and this effect appears to be mediated by nitric oxide and, to some extent, vasoactive intestinal polypeptide (Barthó et al., 2008). Despite these motor effects of TRPV1 stimulation in vitro, the motor activity of the small and large intestine of experimental animals is maintained at a physiological level following functional ablation of capsaicin-sensitive afferent neurons (Barthó et al., 2008). Likewise, no obvious changes in GI motor function have thus far been reported to occur in mice deficient in TRPV1.

Ingestion of capsaicin by humans increases amplitude and velocity of esophageal pressure waves and facilitates secondary peristalsis due to air injection, decreases proximal gastric tone, inhibits phasic contractions of the proximal stomach and inhibits gastric emptying, but does not significantly alter orocecal transit time (Gonzalez et al., 1998; Lee et al., 2004; Chen et al., 2010). The capsaicin-induced improvement of esophageal motility has been observed in healthy volunteers as well as in patients with gastro-esophageal reflux disease (Gonzalez et al., 1998; Grossi et al., 2006; Chen et al., 2010). The relevance of TRPV1 to GI motor control may be most pronounced under pathopysiological conditions when stimulation of TRPV1 on sensory nerve fibers causes local messenger release and activation of sympathetic reflexes. In this way, laparotomy or peritoneal irritation slows GI transit and may even cause ileus (Holzer et al., 1986; Barquist et al., 1996; Zittel et al., 2001). This contention is also borne out by the observation that the gastroparesis associated with experimental colitis is relieved by TRPV1 blockers (De Schepper et al., 2008).

Secretory functions in the alimentary canal are subject to regulation by TRPV1 expressed in neurons as well as epithelial cells. In the human submandibular gland, TRPV1 has been found to activate acinar cells, to stimulate the trafficking of aquaporin-5 to the cell membrane and to cause salivary secretion (Ding et al., 2010; Zhang et al., 2010). Acid- or capsaicin-evoked stimulation of epithelial TRPV1 in the feline and human esophagus induces the formation of platelet-activating factor (Cheng et al., 2009; Ma et al., 2010). Exposure of human isolated antral glands to capsaicin causes release of gastrin and somatostatin, an effect that is thought to be mediated by TRPV1 expressed by gastrin cells, because it is blunted by a chili-rich diet ingested for 3 weeks (Ericson et al., 2009). Distension of the guinea-pig colon (Weber et al., 2001) or exposure of the guinea-pig and human colon to H_2_S (Schicho et al., 2006; Krueger et al., 2010) in vitro evokes a prosecretory effect which depends on activation of TRPV1-expressing sensory nerve fibers, release of substance P and stimulation of cholinergic secretomotor neurons.

#### 2.2.3 Association of TRPV1 with gastrointestinal disease

Upregulation of TRPV1 tissue levels has been observed in a number of inflammatory and functional GI disorders (Table 2). Thus, both erosive and non-erosive reflux diseases are associated with increased levels of TRPV1 in the esophageal mucosa (Matthews et al., 2004; Bhat & Bielefeldt, 2006; Guarino et al., 2010; Shieh et al., 2010). The upregulation of TRPV1 in the mucosa of patients with erosive esophagitis is associated with elevated expression of nerve growth factor and glial cell line-derived neurotrophic factor (Shieh et al., 2010). Idiopathic rectal hypersensitivity and fecal urgency (Chan et al., 2003) and Hirschsprung's disease (Facer et al., 2001) are instances of TRPV1 upregulation in the absence of inflammation. Patients with uninvestigated dyspepsia have been found hypersensitive to intrajejunal capsaicin infusion (Hammer, 2006b), and a proportion of patients with functional dyspepsia are more responsive to ingestion of capsaicin capsules than healthy controls (Hammer et al., 2008). A role of TRPV1 in upper GI pain is also suggested by the antinociceptive effect which prolonged treatment with capsaicin-containing capsules has in healthy volunteers and patients with functional dyspepsia (Bortolotti et al., 2002; Führer & Hammer, 2009). Furthermore, the homozygous G315C polymorphism of the TRPV1 gene has been found to be inversely related to symptom severity in functional dyspepsia patients (Tahara et al., 2010).

Patients suffering from diarrhea-predominant irritable bowel syndrome exhibit hypersensitivity to the painful and burning sensations which chili-containing capsules and food elicit in the GI tract (Gonlachanvit et al., 2009). The TRPV1-positive innervation of the mucosa in the rectosigmoid colon is increased in patients with inflammatory bowel disease, in patients with irritable bowel syndrome as well as in patients with quiescent inflammatory bowel disease who continue to complain of irritable bowel syndrome-like symptoms (Yiangou et al., 2001; Akbar et al., 2008, 2010). Importantly, the density of TRPV1-positive nerve fibers in the rectosigmoid colon correlates with pain severity in both patients with irritable bowel syndrome and patients with symptomatic but quiescent inflammatory bowel disease (Akbar et al., 2008, 2010).

#### 2.2.4 Therapeutic potential of TRPV1 ligands in the digestive system

Recognition of TRPV1 as a multimodal nocisensor, its sensitization by a number of proinflammatory and proalgesic pathways, its upregulation under conditions of hyperalgesia and its apparent implication in experimental colitis have made this TRP channel an attractive target for novel antinociceptive and antiinflammatory drugs (Szallasi et al., 2007; Holzer, 2008a, 2008b; Voight & Kort, 2010). Pharmacologically, the function of TRPV1 can be manipulated by two principal approaches: stimulant/defunctionalizing TRPV1 agonists and TRPV1 antagonists (Roberts & Connor, 2006; Gharat & Szallasi, 2008; Gunthorpe & Szallasi, 2008; Baraldi et al., 2010). The mechanism and result of the two approaches are profoundly different. While TRPV1 antagonists specifically modify the function of the ion channel, stimulant/defunctionalizing TRPV1 agonists target the cellular function of capsaicin-sensitive afferent neurons (Holzer, 1991; Szallasi et al., 2007). The “desensitization” that is brought about by capsaicin, resiniferatoxin or non-pungent TRPV1 ligands reflects “defunctionalization” of the whole afferent neuron expressing TRPV1 for a prolonged period of time.

At present, there is only evidence for a beneficial effect of stimulant/defunctionalizing TRPV1 agonists in GI disease. Acute ingestion of capsaicin has a protective effect against aspirin-induced erosions in the gastric mucosa of human volunteers (Yeoh et al., 1995). In addition, capsaicin can improve esophageal motility in patients with gastro-esophageal reflux disease (Grossi et al., 2006). Prolonged intake of capsaicin appears to desensitize afferent nerve fibers against noxious stimulation of the upper GI tract. Thus, ingestion of capsaicin (0.25 mg) capsules by healthy volunteers 3 times per day for 4 weeks blunts the pain response evoked by duodenal capsaicin administration and balloon distension (Führer & Hammer, 2009). Similarly, treatment of patients suffering from functional dyspepsia with capsaicin-containing capsules for 5 weeks leads to a significant reduction of pain symptoms (Bortolotti et al., 2002). Since there is some information that the prevalence of heartburn symptoms is low in Asian countries in which the intake of chili-rich food is common, it has been proposed that chronic ingestion of capsaicin may be beneficial in patients with gastro-esophageal reflux disease and functional dyspepsia (Gonlachanvit, 2010).

A great deal of effort has been put into developing compounds that block TRPV1 activation in a competitive or noncompetitive manner (Gharat & Szallasi, 2008; Gunthorpe & Szallasi, 2008; Kym et al., 2009; Wong & Gavva, 2009; Voight & Kort, 2010). Some TRPV1 blockers exhibit species differences in their activity and/or act in a stimulus-specific manner, i.e., differentially inhibit the activation of TRPV1 by capsaicin, heat, and acid (Gavva et al., 2005; Lehto et al., 2008). The design of stimulus-specific blockers is of particular importance because a number of TRPV1 antagonists turned out to have a pronounced hyperthermic effect that limits their clinical usefulness (Caterina, 2008; Gavva, 2008; Holzer, 2008b; Romanovsky et al., 2009; Wong & Gavva, 2009). Apart from their thermoregulatory perils (Caterina, 2008), TRPV1 antagonists are liable to impair the perception of potentially injurious heat (Voight & Kort, 2010). Furthermore, blockade of TRPV1 will interfere with the physiological role of this nocicensor in surveying the physical and chemical environment and, if necessary, in initiating protective responses. Such a role is obvious in the GI tract in which capsaicin-sensitive afferent neurons constitute a neural alarm system which helps maintaining mucosal homeostasis in the face of pending injury (Holzer, 1998, 2004; Akiba et al., 2006b). Other caveats are posed by the incomplete analysis of the involvement of TRPV1 in GI disease. For instance, the upregulation of TRPV1 in gut disorders does not necessarily reflect a causal implication of TRPV1 but may equally well mirror an epiphenomenon of the disease process (Holzer, 2008a).

There are several opportunities to design TRPV1 blockers such that they can be specifically targeted at aberrantly expressed or aberrantly operating TRPV1 channels (Holzer, 2008b) while sparing their physiological function. These opportunities include the development of modality-specific TRPV1 blockers, of uncompetitive TRPV1 antagonists, of compounds that selectively address certain TRPV1 splice variants that may be particularly disease-relevant, compounds that prevent TRPV1 sensitization, and compounds that prevent the recruitment and trafficking of TRPV1 to the cell membrane (Gavva, 2008; Holzer, 2008b; Romanovsky et al., 2009). If these approaches turn out to be successful, TRPV1 blockers may enjoy a wide spectrum of usefulness in GI disease which includes, pain, hyperalgesia and inflammation associated with gastro-esophageal reflux disease, inflammatory bowel disease, functional Gi disorders including functional dyspepsia and irritable bowel syndrome, disturbances of GI motor activity, and nausea and emesis.

### 2.3 TRPV2 and TRPV3 channels

#### 2.3.1 Occurrence of TRPV2 and TRPV3 in the alimentary canal

TRPV2 has been localized to rodent DRG and nodose ganglion neurons supplying the GI tract (Caterina et al., 1999; Kashiba et al., 2004; Zhang et al., 2004; Stotz et al., 2008; Zhao & Simasko, 2010; Zhao et al., 2010). In contrast to TRPV1, TRPV2 occurs primarily in medium and large diameter afferent neurons (Zhang et al., 2004). In the rat oral mucosa, TRPV2 is not only present in subepithelial nerve fibers, but also in junctional epithelial cells surrounding each tooth, Langerhans cells, dendritic cells, macrophages and endothelial cells of venules, but not in oral epithelial cells (Shimohira et al., 2009). In the rat and guinea-pig small intestine, TRPV2 is found in nerve fibers within the muscularis and myenteric plexus as well as in myenteric cell bodies (Kashiba et al., 2004; Zhang et al., 2004). In addition, TRPV2 occurs in the β-cells of murine pancreatic islets and in the insulinoma cell line MIN6 (Hisanaga et al., 2009). Immunohistochemistry of human tissues has localized TRPV2 to epithelial cells of the salivary glands, pancreatic duct, stomach, duodenum and colon (Kowase et al., 2002).

TRPV3 is highly expressed by epithelial cells in the nose and tongue (Xu et al., 2006). It likewise occurs in DRG and nodose ganglion neurons (Zhang et al., 2004; Stotz et al., 2008; Zhao & Simasko, 2010; Zhao et al., 2010), but retrograde labeling has failed to identify TRPV3 in vagal afferent neurons supplying the murine stomach (Zhang et al., 2004). However, the muscle and mucosa of the murine stomach and small intestine do contain TRPV3 mRNA (Zhang et al., 2004). In addition, TRPV3 is expressed by superficial epithelial cells of the murine distal colon, but not by superficial epithelial cells of the stomach, duodenum and proximal colon (Ueda et al., 2009).

#### 2.3.2 Functional implications of TRPV2 and TRPV3 in the digestive system

##### 2.3.2.1 Sensory modalities of TRPV2 and TRPV3

In terms of their thermosensory modalities, TRPV2 and TRPV3 belong to the group of thermo-TRP channels because they are activated by specific temperatures (Dhaka et al., 2009). While TRPV2 is typically stimulated by temperatures above 52 °C (Caterina et al., 1999), TRPV3 responds to temperatures in the innocuous physiological range (Bandell et al., 2007). TRPV2 and in particular TRPV3 are sensitive to a variety of chemical entities and—together with TRPA1, TRPM8 and TRPV1—TRPV3 is one of the multimodal spice sensors (Table 1). Noteworthy, TRPV3 is activated by a range of monoterpenoid compounds (Vogt-Eisele et al., 2007) including carvacrol (oregano), thymol (thyme) and menthol (mint), although these compounds are also recognized by other TRP channels such as TRPA1 and TRPM8.

##### 2.3.2.2 Emerging role of TRPV2 and TRPV3 in digestion

The expression of TRPV2 and TRPV3 in the oral cavity, nose and tongue together with their sensory modalities suggests that these TRP channels play a role in probing food for its thermal and chemical qualities. Specific implications of TRPV2 and TRPV3 in the GI tract have not yet been explored. There is evidence, however, that TRPV2 plays a role in the autocrine regulation of pancreatic β-cells, since insulin causes translocation and insertion of TRPV2 into the plasma membrane and the insulin-evoked calcium entry is prevented by TRPV2 inhibition or knockdown (Hisanaga et al., 2009). In addition, the release of insulin induced by a high glucose concentration depends on TRPV2.

#### 2.3.3 Association of TRPV3 with gastrointestinal disease

A study analyzing a possible association between genetic variability (as examined for 392 single-nucleotide polymorphisms) in 43 fatty acid metabolism-related genes and risk for colorectal cancer in 1225 patients with cancer and 2032 controls has shown that TRPV3 is associated with a higher risk for development of colorectal cancer (Hoeft et al., 2010).

### 2.4 TRPV4 channels

#### 2.4.1 Occurrence of TRPV4 in the alimentary canal

In the GI tract, TRPV4 occurs primarily in fibers of extrinsic primary afferent neurons, although some epithelial and other cells have also been reported to stain positively for this TRPV channel subunit. TRPV4 is present in the nodose ganglion, DRG, stomach, small intestine and colon of rodents (Zhang et al., 2004; Grant et al., 2007; Cenac et al., 2008; Brierley et al., 2008; Zhao & Simasko, 2010; Zhao et al., 2010). Retrograde labeling shows that vagal afferent neurons projecting to the murine forestomach contain TRPV4 and that, in these neurons, TRPV4 is coexpressed with TRPV1, TRPV2 and TRPA1 to various degrees (Zhang et al., 2004). Similarly, DRG neurons projecting to the murine pancreas synthesize both TRPV4 and TRPA1 (Ceppa et al., 2010). The expression of TRPV4 in the DRG neurons that project to the murine and human intestine via mesenteric and pelvic nerves exhibits pronounced regional differences (Brierley et al., 2008; Cenac et al., 2008; Sipe et al., 2008). Thus, TRPV4 is particularly abundant in DRG neurons supplying the colon, in which it is coexpressed with CGRP (Brierley et al., 2008; Cenac et al., 2008; Sipe et al., 2008). In the human colon, TRPV4 is found in fine nerve fibers associated with blood vessels in the submucosa and serosa, while the myenteric plexus, the circular and the longitudinal smooth muscles are largely negative (Brierley et al., 2008).

In the murine colon, TRPV4 occurs in brush-bordered epithelial cells, but not in mucus-secreting epithelial cells, as well as in mucosal glial cells and unidentified cells of the submucosal and muscular layers (Cenac et al., 2008; D'Aldebert et al., 2011). TRPV4 is likewise expressed by epithelial cells of the human colon and by the human colon carcinoma cell line Caco-2 (D'Aldebert et al., 2011).

A further location of TRPV4 is in the hepatobiliary tract in which it is expressed by ciliated cholangiocytes, as shown for both the rat, murine and human liver and bile system (Gradilone et al., 2007, 2010). In these cells, TRPV4 occurs specifically in the apical membrane and the cilia which are thought to subserve a mechano- and osmosensory role (Gradilone et al., 2007).

#### 2.4.2 Functional implications of TRPV4 in the digestive system

##### 2.4.2.1 Sensory modalities of TRPV4

The sensory modalities of TRPV4 (Table 1) include strong acidosis, hypotonicity, warmth, mechanical stimuli such as distension of the gut, 5,6-epoxyeicosatrienoic acid (an endogenously formed metabolite of arachidonic acid), and the synthetic phorbol ester 4-α-phorbol 12,13-didecanoate (Güler et al., 2002; Mizuno et al., 2003; Suzuki et al., 2003; Nilius et al., 2007). Although it has been surmised that the mechanosensory function of TRPV4 reflects a secondary transducer role, a patch-clamp study indicates that TRPV4 is a primary detector of mechanical forces (Loukin et al., 2010).

##### 2.4.2.2 Role of TRPV4 in gastrointestinal pain and hyperalgesia

TRPV4 has turned out to play a major role in mechanical pain and hyperalgesia in the colon. TRPV4 agonists excite colonic afferent neurons in the mouse (Sipe et al., 2008) and enhance the mechanosensory responses of colonic serosal and mesenteric afferent nerve fibers, while the mechanosensitivity of these high-threshold afferent nerve fibers is substantially attenuated in TRPV4 knockout mice (Brierley et al., 2008). The TRPV4 agonist-evoked sensitization of colonic afferent nerve fibers to mechanical stimuli is associated with mechanical hyperalgesia, as the visceromotor response to colorectal distension is enhanced (Cenac et al., 2008; Sipe et al., 2008). Vice versa, TRPV4 knockout or intravertebral pretreatment of mice with TRPV4-directed small interfering RNA (siRNA) reduces the visceromotor response to colonic distension in the noxious range (Brierley et al., 2008; Cenac et al., 2008). Taken these findings together, the specific contribution to colonic high-threshold mechanosensory function makes TRPV4 currently the only nociceptor-specific TRP channel in the gut (Blackshaw et al., 2010).

TRPV4 and PAR-2 are colocalized in spinal afferent neurons (Grant et al., 2007; Sipe et al., 2008), in which these two entities interact with each other to cause mechanical hyperalgesia in the murine colon (Cenac et al., 2008; Sipe et al., 2008). Thus, the excitatory effect of TRPV4 stimulation on firing of colonic afferents is increased by a PAR-2 agonist (Sipe et al., 2008), and a subnociceptive dose of a PAR-2 agonist is able to sensitize colonic afferents to a subnociceptive dose of a TRPV4 agonist (Cenac et al., 2008). Activation of PAR-2 likewise evokes discharge of action potentials in colonic afferent nerve fibers and enhances the visceromotor response to colorectal distension. These effects of PAR-2 agonism are absent in TRPV4 knockout mice (Sipe et al., 2008) as well as after pretreatment with TRPV4 siRNA (Cenac et al., 2008).

Based on these findings it is inferred that TRPV4 plays two distinct roles in colonic pain and hyperalgesia. Under basal conditions, TRPV4 is involved in mechanical nociception, and in this capacity is likely to operate as primary mechanodetector. In addition, TRPV4 mediates the mechanical hyperalgesia evoked by PAR-2 agonism. In this instance, TRPV4 seems to act as secondary transducer of PAR-2, a conclusion that is consistent with the observation that this secondary transducer role of TRPV4 is not confined to PAR-2. Thus, 5-HT and histamine can likewise enhance the expression of TRPV4 in DRG neurons projecting to the murine colon and are able to increase the ability of TRPV4 agonism to induce mechanical hyperalgesia (Cenac et al., 2010). Importantly, the colonic hypersensitivity to colorectal distension evoked by 5-HT or histamine is attenuated by TRPV4 siRNA (Cenac et al., 2010).

In DRG neurons projecting to the murine colon, TRPV4 is not only colocalized with PAR-2 but also with PAR-4 (Augé et al., 2009). Unlike PAR-2 agonism, activation of PAR-4 counteracts mechanical pain and hyperalgesia. Thus, the excitation of DRG neurons by PAR-2 and TRPV4 agonists is reduced and the ensuing mechanical hypersensitivity to colonic distension is attenuated by a PAR-4 agonist (Augé et al., 2009).

TRPV4 is also likely to contribute to the pain associated with pancreatitis. Intraductal administration of a TRPV4 agonist to the murine pancreas causes expression of c-Fos in the spinal cord (Ceppa et al., 2010). Deletion of the TRPV4 gene inhibits the input to the spinal cord and the pain behavior associated with experimental pancreatitis due to caerulein (Ceppa et al., 2010).

##### 2.4.2.3 Role of TRPV4 in the gastrointestinal mucosa and hepatobiliary system

Emerging evidence attributes TRPV4 a proinflammatory role in the colonic mucosa. Exposure of Caco-2 cells and the human intestinal epithelial cell line T84 to the TRPV4 agonist 4-α-phorbol 12,13-didecanoate increases the intracellular calcium concentration and causes release of chemokines (D'Aldebert et al., 2011). In keeping with these effects, intraluminal administration of the TRPV4 agonist to the murine colon evokes a transient increase in paracellular permeability and colitis (D'Aldebert et al., 2011). Furthermore, the expression of TRPV4 in epithelial cells of the colon is significantly enhanced in mice with DSS-induced colitis (D'Aldebert et al., 2011).

Hypotonicity causes an increase in the intracellular calcium in cultured murine cholangiocytes and rat intrahepatic bile duct units, a response that is mimicked by a TRPV4 agonist and impaired by TRPV4 knockdown (Gradilone et al., 2007). The hypotonicity-induced activation of cholangiocytes leads to TRPV4-dependent bicarbonate secretion, the main determinant of ductal bile formation. This mechanism operates as well in vivo, since intrabiliary infusion of a TRPV4 agonist increases luminal bicarbonate secretion and bile flow in the rat, this effect being prevented by TRPV4 blockade (Gradilone et al., 2007). In polycystic kidney rats in which mutations in the polycystic kidney and hepatic disease 1 gene cause cholangiocytes to undergo hyperproliferation and to form cysts, TRPV4 is overexpressed and mislocalized in the cholangiocytes, given that TRPV4 is present intracellularly rather than at the apical membrane and the cilia (Gradilone et al., 2010). TRPV4 agonists attenuate the growth of cultured cholangiocytes from polycystic kidney rats and retard the formation of cysts in culture, whereas the growth of cholangiocytes from normal rats is not altered (Gradilone et al., 2010).

#### 2.4.3 Association of TRPV4 with gastrointestinal disease

TRPV4 channelopathies are responsible for a number of hereditary sensory and motor neuropathies as well as diseases with defects in bone development (Nilius & Owsianik, 2010). Thus, mutations of TRPV4 are found in congenital distal spinal muscular atrophy and scapuloperoneal spinal muscular atrophy (two hereditary motor neuropathies), hereditary motor and sensory neuropathy of type 2C (also known as Charcot–Marie–Tooth disease type 2C) (Auer-Grumbach et al., 2010; Deng et al., 2010; Landouré et al., 2010) and other rare motor neuropathies (Zimoń et al., 2010). Diseases with defective bone development such as metatropic dysplasia, brachyolmia, spondylo-epiphyseal dysplasia of Kozlowski type, spondylo-epiphyseal dysplasia of Maroteaux type (pseudo-Morquio syndrome type 2) and parastremmatic dysplasia are also associated with TRPV4 channelopathies (Camacho et al., 2010; Loukin et al., 2010; Nishimura G. et al., 2010).

Although the GI tract is not the prime victim of these diseases, gastro-esophageal reflux has been observed in hereditary sensory neuropathy of type 1B (Auer-Grumbach, 2008). Little is known, however, whether the hereditary sensory neuropathies of types 1A, 1B and 1D (Auer-Grumbach, 2008) impact on GI sensation and nociception, although the available reports do not mention any conspicuous GI disease phenotype.

TRPV4 in sensory neurons is upregulated in inflammatory bowel disease, and serosal blood vessels in tissues with active colitis are more densely innervated by TRPV4-positive nerve fibers than in biopsies from healthy controls (Brierley et al., 2008). Expression of TRPV4 in colonic epithelial cells of mice with experimental colitis is likewise upregulated, while the levels of TRPV4 mRNA in mucosal biopsies taken from patients with inflammatory bowel disease have been found unaltered (D'Aldebert et al., 2011). However, the colonic mucosa of inflammatory bowel disease patients is infiltrated by TRPV4-positive CD45-positive leukocytes which are absent from the mucosa of healthy controls (D'Aldebert et al., 2011).

In patients with polycystic liver diseases (autosomal-recessive polycystic kidney disease and autosomal-dominant polycystic kidney disease) TRPV4 is overexpressed in cholangiocytes, a finding that is reproduced in polycystic kidney rats, a model for autosomal-recessive polycystic kidney disease (Gradilone et al., 2010).

#### 2.4.4 Therapeutic potential of TRPV4 ligands in the digestive system

Taken together, the experimental findings related to TRPV4 function in the digestive system attribute this TRP channel a pathophysiological role in inflammation, pain, hyperalgesia and polycystic liver disease. This inference if supported by the overexpression of TRPV4 in experimental colitis, inflammatory bowel disease and polycystic liver disease. Since TRPV4 appears to be a common mediator of mechanical hyperalgesia in the colon, it presents itself as a novel therapeutic target in patients with abdominal pain. In addition to their antinociceptive and antihyperalgesic potential, TRPV4 blockers may be useful as antiinflammatory drugs. To the contrary, TRPV4 agonists seem to be beneficial in polycystic liver disease in which they can inhibit cholangiocyte proliferation and cyst formation (Gradilone et al., 2010). Indeed, TRPV4 agonists and blockers are already on the horizon (Vincent et al., 2009) and await to be assessed for their therapeutic efficacy and safety.

### 2.5 TRPV5 and TRPV6 channels

#### 2.5.1 Occurrence of TRPV5 and TRPV6 in the alimentary canal

TRPV5 and TRPV6 are expressed in the mucosa of the human and rodent small and large intestine (Clapham et al., 2005; Nijenhuis et al., 2005; Schoeber et al., 2007; Benn et al., 2008; Suzuki et al., 2008; Fukushima et al., 2009; Peleg et al., 2010). While TRPV5 occurs primarily in the kidneys, TRPV6 is predominantly expressed in intestinal epithelial cells and has also been localized to human colon cancer (Caco-2) cells (Schoeber et al., 2007; Fukushima et al., 2009; Bartik et al., 2010). In addition, TRPV5 mRNA occurs in rat nodose ganglion neurons expressing cholecystokinin (CCK) CCK_1_ receptors (Zhao & Simasko, 2010), which are likely to project to the GI tract.

#### 2.5.2 Functional implications of TRPV5 and TRPV6 in the digestive system

TRPV5 and TRPV6 are the most Ca^2+^-selective members of the TRP ion channel family, play an important role in intestinal Ca^2+^ absorption (Clapham et al., 2005; Nijenhuis et al., 2005; Schoeber et al., 2007; Benn et al., 2008; Suzuki et al., 2008), and undergo calcium-induced inactivation via a phospholipase C-mediated pathway (Thyagarajan et al., 2009). The functional roles of TRPV5 and TRPV6 are interconnected with each other, given that TRPV5 knockout mice upregulate their intestinal TRPV6 expression to compensate for the negative Ca^2+^ balance caused by the loss of TRPV5-mediated Ca^2+^ reabsorption in the kidney (Suzuki et al., 2008). In contrast, TRPV6 knockout mice do not display any compensatory mechanism, which results in secondary hyperparathyroidism (Benn et al., 2008; Suzuki et al., 2008).

The expression and function of both TRPV5 and TRPV6 are subject to regulation by dietary conditions and multiple calciotropic hormones (Nijenhuis et al., 2005; Schoeber et al., 2007; Suzuki et al., 2008). Calcium deficiency in the diet is an important factor that increases the expression of TRPV6 in the duodenal mucosa of mice (Ko et al., 2009). Short chain fatty acids, the fermentation products of fructooligosaccharides, likewise increase expression of TRPV6 and calcium absorption in the rat colorectal epithelium as well as in Caco-2 cells (Fukushima et al., 2009). Curcumin, a polyphenol present in turmeric, is a ligand of the nuclear vitamin D receptor, activation of which enhances the expression of TRPV6 mRNA in Caco-2 cells (Bartik et al., 2010).

Parathyroid hormone and 1,25-dihydroxyvitamin D3 are particularly relevant to the expression of TRPV5 and TRPV6 in the intestinal mucosa (Nijenhuis et al., 2005; Schoeber et al., 2007). In chicken intestinal epithelial cells, 1,25-dihydroxyvitamin D3 and parathyroid hormone increase calcium uptake by stimulating a protein kinase A-mediated pathway to release β-glucuronidase, which in turn activates TRPV6 (Khanal et al., 2008). Consistent with this secondary transducer role of TRPV6 is the finding that the 1,25-dihydroxyvitamin D3-induced calcium uptake is abolished by TRPV6 siRNA (Khanal et al., 2008). In normal human duodenal mucosa obtained at endoscopy, TRPV6 expression is likewise increased following exposure to 1,25-dihydroxyvitamin D3 (Balesaria et al., 2009). Although there is a close functional interconnection between vitamin D and intestinal TRPV6, these factors can also operate independently of each other. Thus, the 1,25-dihydroxyvitamin D3-induced intestinal calcium transport in the murine duodenum does not entirely depend on TRPV6, because intestinal calcium uptake also occurs in TRPV6 knockout mice (Benn et al., 2008). In addition, intestinal calcium absorption and duodenal TRPV6 expression are upregulated in pregnant and lactating mice even in the absence of the nuclear vitamin D receptor (Fudge & Kovacs, 2010). Other hormones that impact on intestinal calcium absorption include prolactin which induces TRPV6 mRNA in the rat duodenal epithelium, synergizes with 1,25-dihydroxyvitamin D3 in enhancing TRPV6 expression and stimulates duodenal calcium transport (Ajibade et al., 2010).

Calcium homeostasis is disturbed in patients with Crohn's disease (Huybers et al., 2008). Similarly, heterozygous mice carrying a deletion in the adenine- and uridine-rich elements of the tumor necrosis factor gene, considered to be a model for Crohn's disease, display an increase in bone resorption along with a decrease in the duodenal expression of TRPV6 mRNA (Huybers et al., 2008). TRPV6 in the intestinal epithelium also appears to have a bearing on colon cancer development. Thus, TRPV6 expression is significantly enhanced in the *Citrobacter rodentium*-induced transmissible murine colonic hyperplasia model (Peleg et al., 2010). Whereas in the normal colon TRPV6 is restricted to the apical membrane of absorptive enterocytes, in the hyperplasia model TRPV6 is also distributed to the proliferating zone of the colonic crypts, in which it occurs in the basolateral membrane and perinuclear area of many epithelial cells (Peleg et al., 2010). When the animals are fed with a calcium-rich diet, the overexpression of TRPV6 in the hyperplasia model is reversed.

#### 2.5.3 Association of TRPV6 with gastrointestinal disease

Increased levels of colonic TRPV6 are associated with early-stage colon cancer. Thus, TRPV6 has been found to be overexpressed in 66% of stage I tumors and in 17% of stage II tumors but is barely detectable in stages III and IV tumors (Peleg et al., 2010). These results suggest that aberrant function of TRPV6 is associated with early colon carcinogenesis but not with frank malignancy (Peleg et al., 2010). This inference is supported by experiments with the human colon carcinoma cell line Caco-2, which constitutively express high levels of TRPV6. Treatment of these cells with TRPV6-specific siRNA significantly reduces TRPV6 mRNA levels by about 50%, which is associated with a 40% reduction of cell proliferation and a more than twofold increase in apoptosis (Peleg et al., 2010). Together with experiments in mice with colon crypt hyperplasia (Peleg et al., 2010), these findings indicate that TRPV6 promotes the proliferation of colonic epithelial cells and may be a pathogenetic factor in the early stages of colon cancer development.

#### 2.5.4 Therapeutic potential of TRPV6 ligands in the digestive system

Pharmacological interference with TRPV6 in the digestive system is likely to interfere with calcium absorption and bone mineralization as well as with epithelial cell hyperplasia and early stages of malignancies. Thus, blockade of TRPV6 can be considered as a measure to prevent and inhibit the development of colon cancer, but this effect needs to be balanced against any negative impact on calcium homeostasis.

### 2.6 TRPA1 channels

#### 2.6.1 Occurrence of TRPA1 in the alimentary canal

In the GI tract, TRPA1 occurs in 3 distinct cellular systems: extrinsic primary afferent neurons, intrinsic enteric neurons and endocrine cells of the mucosa.

Primary sensory neurons constitute the most significant source of TRPA1 in the body, given that TRPA1 is abundantly expressed by trigeminal ganglion, nodose ganglion and DRG neurons (Story et al., 2003; Zhang et al., 2004; Kobayashi et al., 2005; Anand et al., 2008; Yang et al., 2008; Brierley et al., 2009; Kondo et al., 2009, 2010; Yu et al., 2009; Cattaruzza et al., 2010; Ceppa et al., 2010; Hondoh et al., 2010; Zhao et al., 2010). In rodent and human DRG neurons, especially in those projecting to the gut, TRPA1 is preferentially expressed by small and medium diameter cell bodies in which it is colocalized with TRPV1 and CGRP (Bautista et al., 2005; Kobayashi et al., 2005; Anand et al., 2008; Brierley et al., 2009; Kondo et al., 2009, 2010; Cattaruzza et al., 2010; Hondoh et al., 2010; Zhao et al., 2010). Similarly, TRPA1 on nodose ganglion neurons in the guinea-pig is primarily associated with small to medium diameter somata and co-expressed with PAR-2 (Yu et al., 2009). In the periphery, TRPA1 has been found in the stomach, small intestine, colon and pancreas of the rat and mouse, the major source being peripheral nerve fibers of DRG and nodose ganglion neurons (Zhang et al., 2004; Penuelas et al., 2007; Brierley et al., 2009; Kondo et al., 2009, 2010; Cattaruzza et al., 2010; Ceppa et al., 2010; Dong Y. et al., 2010). The distribution of TRPA1 in the rodent gut is similar to that in the canine gut, given that TRPA1 is strongly expressed in the stomach, pancreas, small intestine and colon of the dog (Doihara et al., 2009a).

The other sources of TRPA1 in the GI tract are neurons of the human and murine enteric nervous system (Penuelas et al., 2007; Anand et al., 2008) as well as 5-HT-releasing enterochromaffin cells and CCK-releasing endocrine cells of the human, rat and murine GI mucosa (Purhonen et al., 2008; Nozawa et al., 2009). The murine neuroendocrine cell line STC-1, the rat endocrine cell line RIN14B and the human pancreatic endocrine cell line QGP-1, which contain enterochromaffin cell markers, likewise express TRPA1 (Purhonen et al., 2008; Doihara et al., 2009c; Nozawa et al., 2009).

#### 2.6.2 Functional implications of TRPA1 in the digestive system

##### 2.6.2.1 Sensory modalities of TRPA1

TRPA1 was originally identified as a cold-activated channel (Story et al., 2003), a role that later provoked some controversy as TRPM8 is attributed a greater role in this respect than TRPA1 (Kwan et al., 2006; Bandell et al., 2007; Karashima et al., 2009; Knowlton et al., 2010). Most remarkable, however, is that TRPA1 turned out to be a multimodal sensor not only for various spices but also for an extensive list of endogenous and exogenous irritant chemicals including a number of drugs in clinical use (Table 1). Many of these pungent and irritating chemicals are reactive electrophiles which need to be recognized and avoided because of their potential to damage tissues through modification of nucleic acids, proteins and other biomolecules (Kang et al., 2010).

Its sensory modalities (Table 1) place TRPA1 specifically in a position (1) to taste spicy compounds present in mustard, horseradish, wasabi, galangal, black pepper, garlic, onion, cinnamon, ginger, oregano, wintergreen and clove (Table 1), (2) to detect toxic environmental stimuli (Table 1) such as ozone, tear gas, nicotine, formaldehyde, acrolein, iodoacetamide, methyl p-hydroxybenzoate (an antibacterial added to food, pharmaceutical and cosmetic products), irritating drugs (e.g., acetaminophen metabolites, fenamate nonsteroidal antiinflammatory drugs, isoflurane, propofol, dihydropyridines, and clotrimazole), and industrial chemicals (e.g., methyl isothiocyanate, and styrene), and (3) to survey the alimentary canal for potentially deleterious conditions arising from the presence of alkalosis (Fujita et al., 2008), H_2_S, nitric oxide, and oxidative insult products such as 4-hydroxy-2-nonenal, H_2_O_2_, and acetaldehyde (Table 1).

Although there is emerging evidence that TRPA1 contributes to mechanosensation in the gut (Brierley et al., 2009), it appears that TRPA1 does not operate as primary mechanosensor. The slowly adapting currents which indentation of the soma membrane causes primarily in isolectin B_4_-negative DRG neuron cultures are suppressed by the TRPA1 blocker HC-030031 and are absent in TRPA1 knockout mice (Vilceanu & Stucky, 2010). Analysis of TRPA1-transfected HEK293 cells, however, shows that TRPA1 alone is not sufficient to confer mechanical sensitivity (Vilceanu & Stucky, 2010). It would appear, therefore, that TRPA1 functions as a secondary transducer of primary mechanosensors that await to be identified.

##### 2.6.2.2 Sensory function of TRPA1 in the digestive system

In the oral cavity, TRPA1 mediates the taste of many spices (Table 1) and appears to contribute to the stinging and pungent sensations caused by carbon dioxide and carbonated beverages (Wang et al., 2010). Once it enters sensory nerve fibers, carbon dioxide causes intracellular acidification, which in turn activates TRPA1 (Wang et al., 2010).

Many studies have shown that activation of TRPA1 in the esophagus and GI tract causes excitation of primary afferent neurons in the vagus, splanchnic and pelvic nerves. TRPA1 agonists such as allyl isothiocyanate (AITC) excite vagal afferent C-fibers, but not Aδ-fibers, in the guinea-pig esophagus (Yu & Ouyang, 2009) and spinal afferent neurons in the murine colon (Cattaruzza et al., 2010). Accordingly, intracolonic administration of TRPA1 agonists to rodents induces c-Fos expression in the spinal cord (Cattaruzza et al., 2010; Mitrovic et al., 2010) and elicits nociception as deduced from the occurrence of a visceromotor response (Yang et al., 2008) and referred pain (Laird et al., 2001). Intraductal administration of a TRPA1 agonist to the murine pancreas likewise causes expression of c-Fos in the spinal cord (Ceppa et al., 2010). These nociceptive reactions to TRPA1 stimulation in the digestive system are inhibited by the TRPA1 blocker HC-030031 (Mitrovic et al., 2010), by intrathecal pretreatment with a TRPA1 antisense oligodeoxynucleotide (Yang et al., 2008) or by TRPA1 gene knockout (Brierley et al., 2009; Cattaruzza et al., 2010; Ceppa et al., 2010).

TRPA1 is required for normal mechano- and chemosensory function in distinct subsets of vagal, splanchnic and pelvic afferent neurons supplying the murine GI tract (Brierley et al., 2009). Specifically, spike discharges in response to punctate mechanical stimulation in splanchnic afferents with receptive fields in the mesentery and serosa and in pelvic afferents with receptive fields in the mucosa, muscularis and serosa are attenuated in TRPA1 knockout mice, while the responses of pelvic afferents to muscle stretch remain unabated (Brierley et al., 2009). Despite these findings there is at present uncertainty as to whether TRPA1 contributes to normal mechanonociception in the colon, given that the visceromotor response to noxious colorectal distension has been reported to be reduced (Brierley et al., 2009) or to remain unchanged (Cattaruzza et al., 2010) in TRPA1 knockout mice. In contrast, there is pharmacological evidence that in the rat stomach TRPA1 plays a role in mechanical nociception, given that the pseudoaffective pain reaction (contraction of the acromiotrapezius muscle) to gastric distension is attenuated by intrathecal administration of a TRPA1 antisense oligodeoxynucleotide as well as by intrathecal or intraperitoneal injection of HC-030031 (Kondo et al., 2009, 2010).

TRPA1 is colocalized with TRPV1 in many afferent neurons, and there is evidence that the two TRP channels interact with each other in the sensory function of peripheral nerve fibers (Salas et al., 2009; Patil et al., 2010). For instance, the mechanical desensitization which capsaicin induces in splanchnic, but not pelvic, colonic afferents is absent in TRPA1 knockout mice, while the excitatory effect of capsaicin on these nerve fibers remains unaltered by TRPA1 gene deletion (Brierley et al., 2009). The interaction between TRPV1 and TRPA1 is thought to be related to phosphatidyl 4,5-bisphosphate which is a factor relevant to the function of both TRP channels (Brierley et al., 2009).

##### 2.6.2.3 Role of TRPA1 in visceral hypersensitivity

In addition to stimulating afferent neurons, TRPA1 agonists sensitize colonic sensory neurons to mechanical stimulation (Brierley et al., 2009) and enhance the visceromotor response to colorectal distension (Cattaruzza et al., 2010), these effects being prevented by TRPA1 gene knockout or HC-030031. The ability of AITC to sensitize mechanosensitive afferent neurons may be related to its effect to enhance the level of TRPA1 in the colon (Kimball et al., 2007). This argument is supported by a study in which AITC-induced irritation of the colon in newborn mouse pups results in a long-lasting hypersensitivity to colorectal distension as examined in the grown-up animals (Christianson et al., 2010). The enhanced visceromotor response to colorectal distension seen in these experiments is thought to be driven by an early increase in colonic neurotrophin expression, which leads to a permanent increase in the percentage of TRPA1-positive DRG neurons, whereas the number of TRPV1-positive DRG cells remains unaltered (Christianson et al., 2010).

There is substantial evidence that TRPA1 contributes to the GI mechanical hypersensitivity that is associated with GI inflammation and stress. For instance, colitis induced by trinitrobenzene sulfonic acid (TNBS) in rats causes upregulation of TRPA1 in DRG neurons innervating the rat colon and enhances the visceromotor response to colorectal distension, this effect being reduced by intrathecal pretreatment with a TRPA1 antisense oligodeoxynucleotide (Yang et al., 2008). Likewise, the effect of TNBS-induced colitis to enhance spinal neuron activation and the visceromotor pain reaction to colorectal distension is absent in TRPA1 knockout mice (Cattaruzza et al., 2010). This observation is consistent with the ability of TNBS-induced colitis to increase the ability of TRPA1 agonists to sensitize mechanosensitive afferents in the splanchnic and pelvic nerves (Brierley et al., 2009). Apart from GI inflammation, stress is also known to give rise to visceral hypersensitivity. The abdominal withdrawal reflex to colorectal distension, which is enhanced following chronic exposure to water avoidance stress for 10 days, is associated with a significant upregulation of TRPA1 in DRG neurons supplying the colon (Yu et al., 2010), but it has not yet been demonstrated whether TRPA1 is causally involved in stress-evoked visceral hyperalgesia.

Under conditions of inflammation, TRPA1 appears to take over a significant role as secondary transducer of several proinflammatory mediators such as bradykinin and endogenous PAR-2 agonists. Pharmacological and gene knockout experiments indicate that TRPA1 mediates the bradykinin-induced mechanical sensitization of splanchnic afferents innervating the colonic serosa of the mouse (Brierley et al., 2009) and the bradykinin-evoked mechanical hypersensitivity of vagal afferent neurons in the guinea-pig esophagus (Yu & Ouyang, 2009). Importantly, the activation of vagal and splanchnic afferents by bradykinin itself is independent of TRPA1 (Brierley et al., 2009; Yu & Ouyang, 2009). Mast cell activation and activation of PAR-2 are further stimuli that cause mechanical hypersensitivity of vagal afferent neurons in the guinea-pig esophagus via a TRPA1-dependent mechanism (Yu et al., 2009). Similarly, the ability of a PAR-2 agonist to enhance the visceromotor response to colorectal distension is abrogated by TRPA1 gene deletion (Cattaruzza et al., 2010), while the effect of a PAR-2 agonist to stimulate colonic afferents in the splanchnic nerves per se is unaltered in TRPA1 knockout mice (Brierley et al., 2009).

The role of TRPA1 is not limited to mechanical hypersensitivity, as TRPA1 also participates in inflammation-evoked chemical hypersensitivity in the digestive system. Induction of mild colitis with DSS causes colonic hypersensitivity to AITC as revealed by increased expression of c-Fos in the murine spinal cord (Mitrovic et al., 2010). This hyperresponsiveness to colonic TRPA1 stimulation is prevented by HC-030031 as well as by morphine, which suggests that the spinal c-Fos response to colonic TRPA1 stimulation reflects colonic chemonociception (Mitrovic et al., 2010). Similarly, TNBS-induced colitis in the rat amplifies the visceromotor response to intracolonic AITC in a TRPA1 antisense oligodeoxynucleotide-sensitive manner (Yang et al., 2008). TNBS-evoked colitis is associated with an upregulation of TRPA1 in DRG neurons supplying the colon (Yang et al., 2008), an effect that may involve neurotrophic factors formed under conditions of irritation and inflammation (Anand et al., 2008; Christianson et al., 2010).

##### 2.6.2.4 Role of TRPA1 in inflammation within the digestive system

AITC has long been used to elicit and examine neurogenic inflammation (Holzer, 1988), and there is evidence that activation of TRPA1 on visceral sensory neurons gives rise to both vasodilatation (Pozsgai et al., 2010) and inflammation (Kimball et al., 2007; Ceppa et al., 2010). Introduction of excess AITC into the murine colon induces colitis which is associated with an upregulation of TRPA1 and other factors of the intestinal nervous and immune systems (Kimball et al., 2007). Similarly, intraductal administration of an AITC agonist to the murine pancreas causes inflammation and activation of spinal neurons, these effects being markedly attenuated in TRPA1 knockout mice (Ceppa et al., 2010). The pancreatitis evoked by caerulein is likewise ameliorated by TRPA1 deletion, as is the activation of spinal neurons and the pain behavior that accompany pancreatitis (Ceppa et al., 2010). Neurogenic inflammation does not necessarily have a deleterious effect on the tissue, given that AITC has a protective effect against experimentally induced gastric lesions in the rat, an effect that appears to involve endogenous prostaglandins (Matsuda et al., 2007). The inhibitory effect which AITC and icilin have on the restitution of wounded monolayers of the rat gastric epithelial cell line RGM1 does not seem to be mediated by TRPA1 (Hayashi et al., 2008).

##### 2.6.2.5 Role of TRPA1 in the gastrointestinal endocrine system

Stimulation of TRPA1 on the murine neuroendocrine cell line STC-1 causes cell activation and release of CCK (Purhonen et al., 2008), and activation of TRPA1 on rat enterochromaffin cells, on the rat endocrine cell line RIN14B and on the human pancreatic endocrine cell line QGP-1 leads to release of 5-HT (Doihara et al., 2009c; Nozawa et al., 2009). These effects of TRPA1 stimulation may be of functional relevance if the broad sensory spectrum of both TRPA1 and GI endocrine cells and the functional implications of CCK and 5-HT in digestion and GI signaling to the brain are considered. However, these implications of TRPA1 await to be explored and analyzed both in vitro and in vivo.

##### 2.6.2.6 Role of TRPA1 in gastrointestinal motor function

Stimulation of TRPA1 in the gut has distinct effects on GI motor activity, the effects of TRPA1 stimulation being region- and species-dependent. Although intracolonic AITC (2.5%) does not alter compliance of the murine colon in vivo (Cattaruzza et al., 2010), isolated segments of the murine colon, but not small intestine, contract in response to AITC (30–100 μM) and allicin, the response being depressed by tetrodotoxin throughout the colon, whereas atropine inhibits the AITC-induced contraction in the distal colon only (Penuelas et al., 2007). In the guinea-pig ileum, AITC is able to evoke contraction via a mechanism that involves 5-HT and activation of 5-HT_3_ receptors, which suggests that the AITC-induced release of 5-HT from enterochromaffin cells has an impact on the control of intestinal motility (Nozawa et al., 2009).

While the effect of TRPA1 stimulation in the intestine is to facilitate motor activity, gastric emptying in the rat in vivo is delayed by the TRPA1 agonists AITC and cinnamaldehyde through an action that also involves 5-HT and activation of 5-HT_3_ receptors (Doihara et al., 2009b). Administration of TRPA1 agonists to the dog in vivo stimulates motor activity in the gastric antrum and jejunum, and elicits giant migrating contractions in the colon (Doihara et al., 2009a). Activation of TRPA1 also has a prokinetic effect in the murine colon, in which AITC is able to antagonize atonic constipation induced by clonidine as well as spastic constipation induced by loperamide (Kojima et al., 2009). However, the physiological implications of TRPA1 in the nerve circuits and smooth muscle mechanisms governing GI motility remain largely unexplored. Indirect evidence suggests that TRPA1 participates in the cold-induced contraction of the rat colon (Dong Y. et al., 2010), but the physiological relevance of this finding is not yet possible to judge.

#### 2.6.3 Association of TRPA1 with gastrointestinal disease

A point mutation in the S4 transmembrane segment of the TRPA1 gene appears to be responsible for an autosomal-dominant familial episodic pain syndrome that is triggered by fasting and physical stress (Kremeyer et al., 2010). Functional analysis shows that this mutation in the TRPA1 channel is associated with a 5-fold increase in inward current following activation and with enhanced secondary hyperalgesia caused by AITC (Kremeyer et al., 2010). It awaits to be explored whether function-relevant mutations in the TRPA1 gene are relevant to GI disease.

#### 2.6.4 Therapeutic potential of TRPA1 ligands in the digestive system

The properties of TRPA1 as a multimodal sensor for a huge variety of irritant chemicals (Table 1) indicate that TRPA1 blockers could be used to manage chemical irritation and pain. In addition, the emerging pathophysiological implications of TRPA1 suggest that both TRPA1 agonists and blockers hold therapeutic potential in particular disease entities. There is good reason to surmise that TRPA1 blockers could be of particular benefit in inflammation-induced hyperalgesia in the GI tract. TRPA1 blockers may also be of use in pancreatitis (Ceppa et al., 2010) and inflammatory bowel disease. The discovery that activation of TRPA1 by some drugs contributes to their adverse effect profile (Table 1), which may include the alimentary canal, provides a rational basis for developing drugs that lack this unwanted property.

TRPA1 blockers such as HC-030031 have already been characterized, and many other compounds are in the pipeline (Baraldi et al., 2010). The interaction between TRPA1 and TRPV1 in the excitation and/or sensitization of nociceptive nerve fibers (Salas et al., 2009; Patil et al., 2010) hints at the possibility that combined TRPA1 plus TRPV1 blockers may have superior efficacy compared with blockers that target TRPA1 or TRPV1 alone. It awaits to be explored whether interference with TRPA1 disturbs temperature homeostasis, as it has been found with TRPV1 blockers. This limitation cannot be neglected, given that intragastric administration of the TRPA1 agonists AITC and cinnamaldehyde increases thermogenesis and reduces heat diffusion in mice (Masamoto et al., 2009).

Apart from TRPA1 blockers, TRPA1 agonists also may have therapeutic potential. The large variety of TRPA1 agonists available to date (Table 1) can be roughly categorized in two classes: (1) agonists that stimulate TRPA1 via a transient and reversible interaction with the channel, and (2) agonists with an electrophilic carbon or sulfur that bind covalently to cysteine residues of the cytoplasmatic N-terminus of TRPA1 (Baraldi et al., 2010). A specific application of TRPA1 agonists is suggested by their prokinetic effect in the murine colon, which—if translated to humans—could be utilized in the management of both atonic and spastic motor stasis of the gut (Kojima et al., 2009). TRPA1 agonists that will be useful in therapy certainly require optimization in their pharmacological profile, since the utility of the currently available TRPA1 agonists is limited by their irritative effects and high reactivity.

### 2.7 TRPM4 and TRPM5 channels

#### 2.7.1 Expression of TRPM4 and TRPM5 in the alimentary canal

Expression of TRPM4 and, particularly, TRPM5, is limited to taste, epithelial and endocrine cells. TRPM4 transcripts have been identified in pancreatic α- and β-cell lines of rodent origin (Cheng et al., 2007; Marigo et al., 2009), and TRPM5 has been localized to pancreatic islets of the mouse in which it is expressed by the insulin-secreting β cells (Colsoul et al., 2010). TRPM5 abounds on the receptor cells of the lingual taste buds as well as on other chemosensory cells, notably on epithelial taste cells of the gut (Zhang et al., 2003; Bezençon et al., 2007; Kaske et al., 2007; Kokrashvili et al., 2009). Both TRPM4 and TRPM5 mRNA are found in the human digestive system (Fonfria et al., 2006). TRPM5 occurs in the stomach, small intestine and colon of mice and humans, the channel occurring in solitary brush (also termed tufted or caveolated) cells at the surface epithelium of the small and large intestine and in endocrine cells of the duodenal glands (Fonfria et al., 2006; Bezençon et al., 2007, 2008; Kaske et al., 2007; Kokrashvili et al., 2009; Young et al., 2009). Endocrine cells isolated from the rat stomach also stain positively for TRPM5 (Nakamura et al., 2010).

#### 2.7.2 Functional implications of TRPM4 and TRPM5 in the digestive system

Functional evidence suggests that TRPM4 contributes to the membrane depolarization and increase in the intracellular calcium concentration that are relevant to glucose-, arginine vasopressin- and glibenclamide-evoked secretion of insulin from pancreatic β-cells and perhaps glucagon from pancreatic α-cells (Cheng et al., 2007; Marigo et al., 2009). TRPM5 is likewise thought to play a major role in the glucose-dependent release of insulin from pancreatic β-cells (Brixel et al., 2010; Colsoul et al., 2010). Specifically, the effect of glucose to release insulin is significantly reduced in TRPM5 knockout mice, which impairs glucose tolerance (Brixel et al., 2010; Colsoul et al., 2010).

The Ca^2+^-gated TRPM5 channel on the receptor cells of the taste buds in the tongue has been established to contribute to signal transduction in the sweet, umami and bitter tastes (Zhang et al., 2003; Damak et al., 2006). Bitter, sweet, and umami tastants bind to GPCRs which initiate a second-messenger signaling cascade involving activation of phospholipase Cβ2 and hydrolysis of phosphatidylinositol 4,5-bisphosphate to diacylglycerol and inositol trisphosphate. The subsequent increase in the intracellular Ca^2+^ concentration gates TRPM5 and causes depolarization of the taste cells and chemical transmission to gustatory afferent neurons (Zhang Z. et al., 2007). Real life gustatory experiences are often evoked by taste mixtures and determined by multiple interactions between different taste stimuli (Talavera et al., 2008). One such interaction is the suppression of sweet taste transduction by bitter tastants such as quinine. Quinine and its stereoisomer quinidine have been found to block TRPM5 currents and to inhibit the nerve impulses of sucrose-stimulated gustatory nerves in mice (Talavera et al., 2008). Since this quinidine-evoked inhibition of sweet taste signaling is absent in TRPM5 knockout mice, it would appear that TRPM5 plays an important role in bitter–sweet taste interactions (Talavera et al., 2008).

Apart from their occurrence in lingual taste cells, TRPM5 is widely expressed by other chemosensory cells, notably by the tufted epithelial cells of the GI tract (Kaske et al., 2007; Rozengurt & Sternini, 2007). Given that the TRPM5-positive cells of the gut express most genes encoding proteins implicated in sweet, bitter, and umami taste signaling (Bezençon et al., 2008), it would appear that TRPM5 exert taste functions throughout the alimentary canal and contribute to the surveillance of the chemical composition in its lumen. This functional implication is corroborated by the finding that the expression of TRPM5 in the upper small intestine is inversely correlated with the blood glucose concentration in type 2 diabetes patients (Young et al., 2009).

Another task of TRPM5-positive taste cells in the digestive system may be to play local effector roles because they express markers of neuronal and inflammatory cells (Bezençon et al., 2008) and can release biologically active messengers in response to stimulation. Solitary chemosensory cells in the upper small intestine of the murine coexpress TRPM5, β-endorphin, methionine enkephalin and uroguanylin (Kokrashvili et al., 2009). Stimulation of these cells with hyperosmolar solutions or glucose causes release of β-endorphin into the intestinal lumen but not blood serum, an effect that is blunted in TRPM5 knockout mice (Kokrashvili et al., 2009). In contrast, the intestinal release of β-endorphin evoked by acidic solutions or lipid exposure appears to be independent of TRPM5 (Kokrashvili et al., 2009).

#### 2.7.3 Therapeutic potential of TRPM5 ligands in the digestive system

Being a transducer of the sweet taste, TRPM5 has been suggested to have therapeutic potential in the management of obesity and related metabolic dysfunctions, given that taste signaling is a critical determinant of ingestive behavior (Sprous & Palmer, 2010). How efficacious such an approach may be is difficult to judge, however, because deletion of TRPM5 does not completely abolish the preference for sweets and energy-rich food. The reason for this finding is that there are TRPM5-independent transduction mechanisms for glucose tasting (Ohkuri et al., 2009) and sweet and calorie-rich nutrients can directly influence brain reward circuits that control food intake independently of functional taste transduction (de Araujo et al., 2008; Ren et al., 2010).

As TRPM5 appears to be involved in glucose-dependent insulin secretion, TRPM5 dysfunction has been suggested to be a factor in the etiology of some forms of type 2 diabetes (Brixel et al., 2010). If so, normalization of TRPM5 function may be envisaged as a target in diabetes management.

### 2.8 TRPM6 and TRPM7 channels

#### 2.8.1 Occurrence of TRPM6 and TRPM7 in the alimentary canal

TRPM6 and TRPM7 occur in GI epithelial cells and, as is true for TRPM7, in ICCs. In the murine gut, TRPM6 and TRPM7 abound in the brush-border membrane of the colon (Schlingmann et al., 2002; Voets et al., 2004; Fonfria et al., 2006; Groenestege et al., 2006; Rondón et al., 2008a, 2008b; Woudenberg-Vrenken et al., 2010). Furthermore, TRPM7 mRNA and protein are expressed by AGS cells, the most common human gastric adenocarcinoma cell line (Kim et al., 2008). Apart from their association with epithelial cells, TRPM7 is present on ICCs of the myenteric plexus and circular muscle throughout the GI tract of mice and humans (Kim et al., 2005, 2006, 2009).

#### 2.8.2 Functional implications of TRPM6 and TRPM7 in the digestive system

Several data suggest that the formation of functional TRPM6 channels in epithelial cell membranes requires co-expression with TRPM7. There is indeed evidence for the formation of heteromeric ion channels consisting of both TRPM6 and TRPM7 subunits, but there is still controversy as to whether TRPM6 can also function on its own and TRPM7 is required only for trafficking of TRPM6 to the plasma membrane (Schlingmann et al., 2007; Quamme, 2008). TRPM6 and TRPM7 possess an atypical α-kinase domain and are involved in Mg^2+^ homeostasis, given that genetic and functional data indicate that both channel kinases play a critical role in the cellular Mg^2+^ absorption within the GI mucosa (especially in the colon) and in the reabsorption of Mg^2+^ in the kidney (Schlingmann et al., 2007; Quamme, 2008; Woudenberg-Vrenken et al., 2010). Homozygous deletion of TRPM6 in mice is lethal, while heterozygous deletion of TRPM6 results in mild hypomagnesemia, a deficit that cannot be compensated for by a magnesium-rich diet (Woudenberg-Vrenken et al., 2010).

TRPM6 and TRPM7 channel function and colonic Mg^2+^ absorption are in fact controlled by many factors. For instance, the hypomagnesemia associated with dietary Mg^2+^ restriction in mice causes upregulation of TRPM6 expression both in the intestine and kidney (Rondón et al., 2008a). The expression of TRPM6, but not TRPM7, mRNA in the colon is likewise enhanced by a Mg^2+^-rich diet (Groenestege et al., 2006). Feeding of mice with long-chain inulin for 2 weeks, which increases Mg^2+^ absorption and enhances bone stores of Mg^2+^, results in a rise of TRPM6 and TRPM7 expression in the hindgut, while TRPM7 in the kidney is down-regulated (Rondón et al., 2008b).

Mg^2+^ is critical for the growth and survival of certain cancer cells, notably the human gastric adenocarcinoma cell line AGS (Kim et al., 2008). Blockade of TRPM7 channels with La^3+^ or suppression of TRPM7 expression by siRNA inhibits the growth and survival of AGS cells (Kim et al., 2008).

In addition to its epithelial function, TRPM7 plays a role in controlling intestinal motor activity. Electrophysiological, molecular biological, and immunohistochemical evidence indicates that TRPM7 participates in the nonselective cation current that underlies the pacemaker activity of ICCs in the GI tract (Kim et al., 2006). Thus, the electrophysiological and pharmacological properties of the nonselective cation current in ICCs of the mouse are identical to those of TRPM7, and the pacemaker activity of ICCs is inhibited by TRPM7-specific siRNA (Kim et al., 2005). TRPM7 expressed on ICCs is also likely to contribute to pacemaker activity in the human gut, since slow waves in the isolated muscle of the human small and large intestine are attenuated by blockade of TRPM7 channels with La^3+^ (Kim et al., 2009).

#### 2.8.3 Association of TRPM6 with gastrointestinal disease

Non-functional mutations of TRPM6 are responsible for a primary disorder termed hypomagnesemia with secondary hypocalcemia (Schlingmann et al., 2002; Voets et al., 2004). The expression of TRPM6 mRNA in the intestinal epithelium is reduced in patients suffering from cholera, a change that is thought to counteract the secretory response to the infection (Flach et al., 2007).

#### 2.8.4 Therapeutic potential of TRPM7 blockers in the digestive system

The ability of TRPM7 channel blockade to inhibit the growth and survival of AGS cells suggests TRPM7 to be a potential target for the treatment of gastric cancer (Kim et al., 2008).

### 2.9 TRPM8 channels

#### 2.9.1 Occurrence of TRPM8 in the alimentary canal

TRPM8 is distributed to several cell types in the digestive system. Being menthol-sensitive, the TRPM8 subunit is expressed in the papillae of the tongue as well as by a distinct population of primary afferent neurons originating in the nodose-petrosal ganglion and DRG (McKemy et al., 2002; Peier et al., 2002; Zhang et al., 2004; Kobayashi et al., 2005; Hondoh et al., 2010; Zhao et al., 2010). Unlike that of TRPA1, the colocalization of TRPM8 with TRPV1 in sensory neurons is limited (Bautista et al., 2005; Kobayashi et al., 2005; Kondo et al., 2009; Hondoh et al., 2010; Zhao et al., 2010). Within the GI tract, TRPM8 has been localized to the muscle of the rat stomach and colon (Mustafa & Oriowo, 2005) as well as to the liver (Fonfria et al., 2006). The expression of TRPM8 in the murine stomach and small intestine remains controversial, given that both positive (Zhang et al., 2004) and negative (Penuelas et al., 2007) results have been reported.

#### 2.9.2 Functional implications of TRPM8 in the digestive system

TRPM8 is activated by temperatures in the cool to cold range and makes an important contribution to cold-induced pain (Bautista et al., 2007; Colburn et al., 2007; Dhaka et al., 2007; Knowlton et al., 2010). Apart from low temperatures, TRPM8 is stimulated by various chemical entities including menthol, icilin, geraniol, L-carvone, isopulegol and linalool (Table 1). These sensory modalities of TRPM8 are likely to explain the refreshing taste of menthol. Also keeping with this property is the finding that intragastric administration of the TRPM8 agonist menthol induces thermogenesis in mice (Masamoto et al., 2009).

Expressed by extrinsic primary afferent neurons and intrinsic systems within the GI tract, TRPM8 may play a chemosensory role in the alimentary canal. This contention is supported by the observation that the mixed TRPA1/TRPM8 agonist icilin is able to stimulate murine nodose ganglion neurons in culture (Zhang et al., 2004). However, systematic studies of the chemosensory role of TRPM8-bearing primary afferent neurons in the gut have not yet been reported, and the precise functional implications of TRPM8 in the digestive system await to be explored. In the murine colon, menthol induces a long lasting relaxation of smooth muscle, which remains unaffected by tetrodotoxin (Penuelas et al., 2007). It is questionable if this motor effect of menthol is due to activation of TRPM8 because the presence of TRPM8 in the murine gut has not unequivocally been established (Zhang et al., 2004; Penuelas et al., 2007). Whether the cooling-induced contraction of the rat gastric fundus (Mustafa & Oriowo, 2005) and guinea-pig ileum (Holzer & Lembeck, 1979) involves TRPM8 has not conclusively been investigated.

### 2.10 TRPP2 channels

#### 2.10.1 Occurrence of TRPP2 in the alimentary canal

The polycystin TRP channel TRPP2 (also known as polycystic kidney disease-like ion channel, PKD2L1) and the related PKD1 polycystin L3 (PKD1L3) are expressed by a select class (type III) of taste chemoreceptor cells in the murine tongue, which are distinct from those mediating sweet, umami and bitter tastes (Huang et al., 2006; Ishimaru et al., 2006; LopezJimenez et al., 2006; Kataoka et al., 2008; Chandrashekar et al., 2009; Kawaguchi et al., 2010). While TRPP2 has been reported to occur in the circumvallate, foliate and fungiform papillae of the tongue and in the palate, PKD1L3 has been localized to the circumvallate and foliate papillae only (Huang et al., 2006; Ishimaru et al., 2006). PKD1, the gene that is mutated in ~85% of autosomal dominant polycystic kidney disease patients, has been described to occur in many tissues including the intestine (Geng et al., 1997).

#### 2.10.2 Functional implications of TRPP2 in the digestive system

Although several members of the TRPV, TRPA and TRPC channel subfamilies are sensitive to alterations in extracellular pH (Holzer, 2009), TRPP2 (PKD2L1) is the TRP channel involved in sour taste transduction (Chandrashekar et al., 2006; Ishimaru & Matsunami, 2009). In order to form functional channels, TRPP2 needs to associate as heteromer with related proteins of the PKD1 polycystin family such as PKD1L3 (Ishimaru et al., 2006; Kawaguchi et al., 2010). The PKD1 polycystins are not included in the TRP channel family because they are large proteins with a very long N-terminal extracellular domain and 11 transmembrane domains that include a 6-transmembrane TRP-like channel domain at the C terminus (Clapham et al., 2005).

Both genetic and functional studies corroborate that TRPP2 plays an important role as sour taste receptor (Huang et al., 2006; Ishimaru et al., 2006; LopezJimenez et al., 2006; Kataoka et al., 2008; Kawaguchi et al., 2010). In the taste buds of the murine tongue, TRPP2 is accumulated at the taste pore region where taste chemicals are detected. Coexpression of TRPP2 and PKD1L3 is necessary for the positioning of functional sour taste receptors at the cell surface, which is the case in the circumvallate and foliate papillae of the murine tongue (Huang et al., 2006; Ishimaru et al., 2006; Chandrashekar et al., 2009; Kawaguchi et al., 2010). In native circumvallate taste cells expressing TRPP2 the pH threshold for acid-induced increases in the intracellular calcium concentration is around 5.0 (Kawaguchi et al., 2010). Targeted ablation of TRPP2-expressing taste receptor cells results in a specific and total loss of sour taste transduction, whereas responses to sweet, umami, bitter and salty tastants remain unchanged (Huang et al., 2006). The relevance of PKD1, which occurs in the intestine (Geng et al., 1997), to GI function remains unexplored.

The TRPP2-expressing sour taste cells also appear to contribute to the taste of carbonation. While the principal CO_2_ taste sensor is carbonic anhydrase-4 (Chandrashekar et al., 2009), it remains to be determined whether TRPP2 plays a role in the downstream signaling of the carbonation taste.

### 2.11 TRPC channels

#### 2.11.1 Occurrence of TRPC in the alimentary canal

Distinct members of the classical or canonical TRP channels (TRPC) are widely distributed in the GI tract, the cellular sources being vagal afferent neurons, enteric neurons, smooth muscle cells, ICCs and epithelial cells. TRPC1, TRPC3, TRPC5 and TRPC6 have been localized to nodose ganglion neurons in which TRPC1 and TRPC6 are, at least in part, coexpressed with TRPV1 and TRPA1. The evidence for this colocalization rests on the finding that systemic pretreatment of rats with a neurotoxic dose of capsaicin causes a loss of TRPV1, TRPA1, TRPC1 and TRPC6 from vagal afferent neurons (Zhao et al., 2010). In the mouse DRG, mRNA for all 7 types of TRPC subunits (TRPC1–TRPC7) has been found, with TRPC1, TRPC3 and TRPC6 being the most abundant (Elg et al., 2007).

All 7 types of TRPC subunits have likewise been identified in the murine stomach (Lee et al., 2003; So & Kim, 2003). TRPC6 is expressed in human esophageal and gastric epithelial cells (Cai et al., 2009; Shi et al., 2009), while TRPC1 and TRPC5 have been localized to intestinal epithelial cells (Rao et al., 2006). In addition, TRPC4, TRPC6 and TRPC7 as well as splice variants of TRPC4 (TRPC4-α and TRPV-β) and TRPC7 are present in GI and vascular smooth muscle of the murine and canine alimentary canal, TRPC4 being the most abundant subunit (Walker et al., 2001, 2002; Torihashi et al., 2002; Lee et al., 2005; Kim et al., 2006; Tsvilovskyy et al., 2009). TRPC1, TRPC3, TRPC4, TRPC5 and TRPC6 mRNA have likewise been localized to circular muscle cells of the human esophageal body and lower esophageal sphincter, the levels of TRPC3 and TRPC4 mRNA being higher in the sphincter than in the body (Wang et al., 2003).

The guinea-pig enteric nervous system expresses TRPC1, TRPC3, TRPC4 and TRPC6, which are differentially distributed to the myenteric and submucosal plexuses (Liu et al., 2008). While TRPC1 abounds in myenteric neurons with cholinergic and nitrergic phenotypes as well as in submucosal cholinergic and noncholinergic secretomotor neurons, TRPC3 is restricted to submucosal neurons containing neuropeptide Y (Liu et al., 2008). TRPC4 and TRPC6 are likewise preferentially expressed by noncholinergic secretomotor neurons of the submucosal plexus (Liu et al., 2008).

#### 2.11.2 Functional implications of TRPC in the digestive system

The abundant distribution of TRPC channels to vagal afferent neurons, enteric neurons, smooth muscle cells, ICCs and epithelial cells in the gut points to multiple functional implications of this class of TRP channels. However, their role in the neural control of digestive functions has not yet been examined. There is, however, increasing evidence that TRPC channels are involved in signal transduction within the epithelium and smooth muscle of the GI tract. In addition, distinct TRPC channels play a physiological role in GI epithelial cell homeostasis and in the development of malignancies.

The phenotype of TRPC1 knockout mice is characterized by a pronounced impairment of neurotransmitter-stimulated salivary gland fluid secretion, which suggests that TRPV1 is relevant to the secretory process in salivary gland acinar cells (Liu et al., 2007). In human colonic epithelial cells TRPC1 is involved in the signal transduction of the extracellular calcium-sensing receptor which is relevant to both intracellular calcium oscillation and inhibition of proliferation (Rey et al., 2010). Wounding of the mucosa causes translocation of stromal interaction molecule-1 to the plasma membrane, which in turn activates TRPC1-mediated calcium influx and promotes intestinal epithelial restitution (Rao et al., 2010). Induced TRPC1 expression facilitates, while inhibition of its expression by TRPC1-directed siRNA inhibits epithelial cell migration after wounding (Rao et al., 2006, 2010). In addition, induced TRPC1 expression sensitizes intestinal epithelial cells to apoptosis via calcium influx-mediated inhibition of nuclear factor-κB activity (Marasa et al., 2006, 2008).

TRPC1 and TRPC6 play a role in pancreatic and GI cancer development. In the pancreatic cancer cell line BxPc3, TRPC1 is a transducer of the action of tumor growth factor-β to stimulate cell motility, a process that is of relevance to tumor cell invasion (Dong H. et al., 2010). TRPC6, which can be activated both by receptor tyrosine kinases and GPCRs, appears to have a role in human esophageal and gastric cancer development (Cai et al., 2009; Shi et al., 2009). Treatment of the human gastric cancer cell lines AGS and MKN45 with the TRPC channel blocker SKF-96,365 arrests the cell cycle in the G2/M phase and suppresses the formation of gastric tumors in nude mice (Cai et al., 2009). Inhibition of TRPC6 likewise arrests the cell cycle of human esophageal squamous carcinoma cells in the G2 phase and inhibits tumor formation in nude mice injected with esophageal squamous carcinoma cells (Shi et al., 2009).

In the exocrine pancreas, TRPC3-mediated calcium influx is attributed an adverse role in the acute inflammation induced by supramaximal stimulation or exposure to toxic bile acids and palmitoleic acid ethyl ester (Kim M.S. et al., 2009). Consequently, deletion of TRPC3 has been found to attenuate experimentally evoked acute pancreatitis (Kim M.S. et al., 2009).

TRPC channels have been proposed to participate in intestinal pacemaker activity of ICCs which through electrical coupling drive slow wave activity in intestinal smooth muscle. The pacemaker current that initiates each slow wave derives from a Ca^2+^-inhibited, voltage-independent, nonselective cation channel in the ICCs. This channel exhibits properties similar to that reported for TRPC channels, notably TRPC4 (Walker et al., 2002). However, slow waves in intestinal smooth muscle remain unchanged in TRPC4 knockout mice (Lee et al., 2005). Thus, there is no conclusive evidence that TRPC channels, notably TRPC4, play a physiological role in the pacemaker activity of ICCs.

In contrast, distinct TRPC channels have been shown to operate as downstream transducers (effectors) of stimulated GPCRs such as muscarinic acetylcholine receptors. This mechanism has been suggested to apply to TRPC4, TRPC5 and TRPC6 which are activated by cholinergic stimulation of ICCs and smooth muscle in the murine stomach and intestine (Lee et al., 2003, 2005; Kim et al., 2006, 2007; Tsvilovskyy et al., 2009). Based on electrophysiological and pharmacological similarities, both TRPC4 (Lee et al., 2005) and TRPC5 (Lee et al., 2003) have originally been proposed to mediate the nonselective cation current evoked by carbachol in the murine stomach. A role of TRPC4 has been firmly established by the finding that the nonselective cation current evoked by muscarinic receptor stimulation is abolished by genetic deletion of TRPC4 (Lee et al., 2005; Kim et al., 2006).

Further studies using both TRPC4 and TRPC6 knockout mice indicate that TRPC4 underlies more than 80% of the muscarinic receptor-induced cation current in intestinal smooth muscle, while the residual current is mediated by TRPC6 (Tsvilovskyy et al., 2009). Following TRPC4 knockout, the carbachol-induced membrane depolarization and the atropine-sensitive contraction elicited by acetylcholine release from excitatory motor neurons are inhibited, these effects being aggravated by additional deletion of TRPC6. In addition, intestinal transit is slowed down in mice lacking TRPC4 and TRPC6 (Tsvilovskyy et al., 2009). From these studies it is evident that TRPC4 and TRPC6 couple muscarinic acetylcholine receptors to depolarization and contraction of intestinal smooth muscle. This is true for both M2 and M3 muscarinic receptors which use different second messenger mechanisms owing to their coupling to G_i/o_ and G_q/11_ signaling pathways, respectively (Tsvilovskyy et al., 2009).

#### 2.11.3 Association of TRPC6 with gastrointestinal disease

An implication of TRPC6 in human GI motor control is exemplified by the observation that a single nucleotide polymorphism in the promoter region of the TRPC6 gene and a missense variant in exon 4 of the TRPC6 gene may contribute to infantile hypertrophic pyloric stenosis (Everett et al., in press). TRPC6 appears to have a role in cancer development, given that there is a marked upregulation of TRPC6 expression in esophageal squamous cell carcinoma (Shi et al., 2009) and in epithelial cells of human gastric cancers (Cai et al., 2009).

#### 2.11.4 Therapeutic potential of TRPC blockers in the digestive system

Cholinergic contraction of smooth muscle is a key determinant of GI motility. Recognition that TRPC4 and, to a minor degree, TRPC6 are transducers of the depolarizing action of M2 and M3 muscarinic acetylcholine receptors in GI smooth muscle raises the possibility to attenuate cholinergic motor effects while sparing other cholinergically controlled tissue functions. This opportunity would be particularly useful if pathological hypermotility in the GI tract is shown to be due to exaggerated TRPC4 and TRPC6 transduction (Ambudkar, 2009).

Blockade of TRPC1 and TRPC6 also holds considerable potential in GI oncology, given that TRPC1 may promote pancreatic cancer cell invasion (Dong H. et al., 2010) and TRPC6 seems to play a significant role in human esophageal and gastric cancer development (Cai et al., 2009; Shi et al., 2009).

## 3 Conclusions

Distinct TRP channels have emerged as pleiotropic signal transducers in the digestive tract. They play physiological roles in taste, chemo- and mechanosensation, thermoregulation, pain and hyperalgesia, epithelial cell function and homeostasis, control of motility by neurons, ICCs and muscle cells, vascular function, and cancer development (Fig. 2). While the implications of some TRP channels, notably TRPA1, TRPC4, TRPM5, TRPM6, TRPM7, TRPV1, TRPV4, and TRPV6, have been elucidated to a considerable degree, the relevance of other TRP channels to digestive function awaits to be explored. The polymodal chemo- and mechanosensory function of TRPA1, TRPM5, TRPV1 and TRPV4 is of particular pathophysiological relevance, but there are several other aspects of GI function that are under the critical control of TRP channels, such as Ca^2+^ and Mg^2+^ absorption, transduction of muscarinic acetylcholine receptor activation to smooth muscle contraction, and cancer development (Fig. 2).

TRP channels in the digestive system can operate (1) as molecular sensors (detectors or primary transducers) of chemical and physical stimuli, (2) as downstream or secondary transducers (or effectors) of cell activation induced by GPCRs, receptor tyrosine kinases or ion channels, and (3) as ion transport channels (Fig. 1). While some TRP channels operate only in one of these capacities, other TRP channels can act both as primary and secondary transducers. This dual role is seen with TRPA1, TRPV1 and TRPV4 which, on the one hand, are detectors of thermal, chemical and mechanical stimuli and, on the other hand, mediate cell responses to other stimuli, notably distension and proinflammatory mediators (Blackshaw et al., 2010). A secondary transducer role is also typical of TRPM5 in its taste function and of TRPC4 in its role to transduce acetylcholine-evoked contraction.

Pathophysiologically, TRP channels participate in many disturbances of GI function (Fig. 3). Changes in TRP channel expression and function are associated with GI inflammation, hyperalgesia and other disease identities, some of which are due to specific TRP channelopathies (Nilius et al., 2007). These implications have raised enormous interest in the therapeutic exploitation of TRP channels as new drug targets in GI disease. As a result, extensive drug development programs have been initiated, especially with regard to TRPV1, TRPA1 and TRPV4 channel blockers (Gharat & Szallasi, 2008; Gunthorpe & Szallasi, 2008; Kym et al., 2009; Vincent et al., 2009; Wong & Gavva, 2009; Baraldi et al., 2010; Voight & Kort, 2010). Fig. 4 summarizes the potential indications of TRP channel agonists and antagonists in the management of GI diseases and disorders. While the results of many preclinical tests involving TRP channel agonism, antagonism, knockdown or knockout nourish these expectations, clinical proof-of-concept data are not yet available, except for TRPV1 desensitization strategies. In the absence of conclusive information it is not possible to predict which TRP channels are the most promising drug targets. The translation from the preclinical validation to the clinical application of TRP channel-directed drugs is liable to meet many obstacles (Hicks, 2006; Storr, 2007; Holzer, 2008a, 2008b; Wong & Gavva, 2009), given that TRP channels are widely distributed throughout the body and play many important physiological roles within and outside the GI system (Wu et al., 2010). The challenge, therefore, is to design TRP channel-directed drugs with a pharmacological profile that limits their action to the alimentary canal and specifically addresses the pathological functions of TRP channels while the physiological implications are spared (Holzer, 2008b).

## Figures and Tables

**Table 1:** Overview of TRP channels with a chemosensory role in the alimentary canal.

TRP channel	Sensory modalities in general	Select chemical agonists	References (regarding chemical agonists)
TRPV1	Heat (>42 °C)	*Spices*	
	Pungent spices	Allicin (garlic, onion)	Macpherson et al., 2005
	Salt taste	Allyl isothiocyanate (mustard, horseradish, wasabi)	Everaerts et al., 2011
	Acidosis	Camphor^a^	Xu et al., 2005
	Alkalosis	Cannabidiol (*Cannabis sativa*)	Bisogno et al., 2001
	Chemesthesis	Capsaicin (red pepper, *Capsicum* ssp.)	Caterina et al., 1997
	Chemical pain	Citral (lemongrass)	Stotz et al., 2008
	Distension	Eugenol (clove)	Bandell et al., 2007
		Evodiamine (*Evodia rutaecarpa*)	Pearce et al., 2004
		Geraniol (citronella grass, *Geranium* ssp.)	Stotz et al., 2008
		Gingerol (ginger)	Bandell et al., 2007
		Isovelleral (*Lactarius vellereus*)	Szallasi et al., 1996; Ralevic et al., 2003
		6-Paradol (Sichuan pepper)	Riera et al., 2009
		Piperine and other black pepper constituents (dehydropipernonaline, isochavicine, isopiperine, piperanine, pipernonaline, piperolein A and B, retrofractamide C)	Andrè et al., 2006; Okumura et al., 2010
		Polygodial (*Polygonum hydropiper*)	Szallasi & Blumberg, 1999
		6-Shogaol (Sichuan pepper)	Riera et al., 2009
		Zingerone (ginger)	Bandell et al., 2007
		*Natural toxins*	
		Brevetoxin (ciguatera and shellfish toxin)	Cuypers et al., 2007
		Gambierol (ciguatera and shellfish toxin)	Cuypers et al., 2007
		Resiniferatoxin (*Euphorbia resinifera*)	Bandell et al., 2007
		Vanillotoxin DkTx (Earth Tiger tarantula venom)	Bohlen et al., 2010
		Vanillotoxins VaTx1, VaTx2, VaTx3 (Trinidad Chevron tarantula venom)	Cromer & McIntyre, 2008
		*Endogenous and exogenous chemicals*	
		Acesulfame-K	Riera et al., 2007
		Acid (pH < 6)	Tominaga et al., 1998
		2-Aminoethoxydiphenyl borate	Hu et al., 2004
		Ammonia (pH > 8)	Dhaka et al., 2009
		Anandamide	Zygmunt et al., 1999
		Clotrimazole	Meseguer et al., 2008
		CuSO_4_	Riera et al., 2007
		Ethanol	Trevisani et al., 2002
		FeSO_4_	Riera et al., 2007
		12-(S)-Hydroperoxy eicosatetraenoic acid	Hwang et al., 2000
		15-(S)-Hydroperoxy eicosatetraenoic acid	Hwang et al., 2000
		Leukotriene B_4_	Hwang et al., 2000
		N-arachidonoyl-dopamine	Huang et al., 2002
		Nitric oxide	Miyamoto et al., 2009
		Nitro-oleic acid (antiinflammatory nitric oxide derivative)	Sculptoreanu et al., 2010
		N-oleoyldopamine	Chu et al., 2003
		Oleoylethanolamide	Wang et al., 2005
		Polyamines (putrescine, spermidine, spermine)	Ahern et al., 2006
		Propofol	Fischer et al., 2010
		Saccharin	Riera et al., 2007
		ZnSO_4_	Riera et al., 2007
TRPV2	Heat (>52 °C) Hypotonicity	*Natural compounds*	
		Cannabidiol (*Cannabis sativa*)	Qin et al., 2008
		Δ^9^-Tetrahydrocannabinol (*Cannabis sativa*)	Qin et al., 2008
		*Synthetic chemicals*	
		2-Aminoethoxydiphenyl borate	Hu et al., 2004; Stotz et al., 2008
		Probenecid	Bang et al., 2007b
TRPV3	Warmth (22–40 °C)	*Spices*	
	Spices	(+)-Borneol (Borneo camphor)	Vogt-Eisele et al., 2007
	Chemesthesis	Camphor^a^	Macpherson et al., 2006; Vogt-Eisele et al., 2007
	Chemical pain	Carvacrol (oregano)	Xu et al., 2006; Vogt-Eisele et al., 2007
		Carveol (spearmint oil)	Vogt-Eisele et al., 2007
		Citral (lemongrass)	Stotz et al., 2008
		Dihydrocarveol (caraway and other plants)	Vogt-Eisele et al., 2007
		Eugenol (clove)	Xu et al., 2006
		Geraniol (citronella grass, Geranium ssp.)	Stotz et al., 2008
		Menthol (mint)	Macpherson et al., 2006
		Thymol (thyme)	Xu et al., 2006; Vogt-Eisele et al., 2007
		Vanillin (vanilla)	Xu et al., 2006
		*Synthetic chemicals*	
		2-Aminoethoxydiphenyl borate	Hu et al., 2004; Stotz et al., 2008
		Ethyl vanillin	Xu et al., 2006
		6-Tert-butyl-*m*-cresol	Vogt-Eisele et al., 2007
TRPV4	Warmth (>25–34 °C)	*Endogenous and exogenous chemicals*	
	Acidosis	Acid (pH < 6)	Suzuki et al., 2003
	Hypotonicity	Bisandrographolide A (*Andrographis paniculata*)	Smith et al., 2006
	Distension	Citrate	Suzuki et al., 2003
		5′,6′-Epoxy eicosatrienoic acid	Watanabe et al., 2003
		4α-Phorbol 12,13-didecanoate	Watanabe et al., 2002
TRPM5	Sweet, umami and bitter taste	*Sweet, umami and bitter tastants*	Zhang et al., 2003; Damak et al., 2006; Kaske et al., 2007
TRPM8	Cold (<25 °C)	*Spices*	
	Spices	L-Carvone (*Mentha spicata*)	Bandell et al., 2007
	Cold pain	Citral (lemongrass)	Stotz et al., 2008
	Chemesthesis	Eucalyptol (*Eucalyptus polybractea*)	Behrendt et al., 2004
	Chemical pain	Eugenol (clove)	Behrendt et al., 2004
		Geraniol (citronella grass, *Geranium* ssp.)	Behrendt et al., 2004; Stotz et al., 2008
		Isopulegol (*Mentha pulegium*)	Bandell et al., 2007
		Linalool (*Onagraceae* ssp.)	Behrendt et al., 2004
		Menthol (mint)^b^	McKemy et al., 2002; Peier et al., 2002
		*Synthetic chemical*	
		Icilin	McKemy et al., 2002
TRPP2	Sour taste	*Acid*	Chandrashekar et al., 2006; Huang et al., 2006; Ishimaru et al., 2006
TRPA1	Cold (<17 °C)	*Spices*	
	Pungent spices	1′-Acetoxychavicol acetate (galangal)	Narukawa et al., 2010
	Alkalosis	Allicin (garlic, onion)	Bautista et al., 2005; Macpherson et al., 2005
	Chemesthesis	Allyl isothiocyanate (mustard, horseradish, wasabi)	Story et al., 2003; Jordt et al., 2004
	Chemical pain	Benzyl isothiocyanate (yellow mustard)	Jordt et al., 2004
	Distension	Carvacrol (oregano)	Xu et al., 2006; Lee et al., 2008
		Cinnamaldehyde (cinnamon)^c^	Bandell et al., 2004
		Citral (lemongrass)	Stotz et al., 2008
		Diallyl disulfide (garlic, onion)	Bautista et al., 2005; Macpherson et al., 2005
		Eugenol (clove)	Bandell et al., 2004
		Geraniol (citronella grass, Geranium ssp.)	Stotz et al., 2008
		Gingerol (ginger)	Bandell et al., 2004
		Isopropyl isothiocyanate (*Nasturtium* seeds)	Jordt et al., 2004
		Linalool (Sichuan pepper)	Riera et al., 2009
		Methyl isothiocyanate (*Capparis spinosa*)	Jordt et al., 2004
		Methyl salicylate (wintergreen)	Bandell et al., 2004
		6-Paradol (Sichuan pepper)	Riera et al., 2009
		Phenylethyl isothiocyanate (Brussels sprouts)	Jordt et al., 2004
		Piperine and other black pepper constituents (isochavicine, isopiperine, piperanine, piperolein A and B)	Okumura et al., 2010
		6-Shogaol (Sichuan pepper)	Riera et al., 2009
		Thymol (thyme)	Lee et al., 2008
		*Natural toxin*	
		GsMTx-4 (tarantula toxin)	Hill & Schaefer, 2007
		*Endogenous and exogenous chemicals*	
		Acetaldehyde	Bang et al., 2007a, 2007b
		Acrolein	Bautista et al., 2006
		2-Aminoethyl methanethiosulfonate	Macpherson et al., 2007a
		Ammonia	Fujita et al., 2008
		Bradykinin	Bandell et al., 2004; Bautista et al., 2006
		Carbon dioxide	Wang et al., 2010
		Croton aldehyde (cigarette smoke)	Andrè et al., 2008
		Cyclopentenone prostaglandin metabolites	Andersson et al., 2008; Materazzi et al., 2008; Maher et al., 2008
		Farnesyl thiosalicylic acid	Maher et al., 2008
		Formaldehyde	Macpherson, Xiao, et al., 2007; McNamara et al., 2007
		H_2_S	Streng et al., 2008
		H_2_O_2_ (oxidative insult)	Andersson et al., 2008; Bessac et al., 2008; Sawada et al., 2008
		4-Hydroxy-2-nonenal (oxidative insult)	Macpherson et al., 2007b; Trevisani et al., 2007
		Hypochlorite (oxidative insult)	Bessac et al., 2008
		Icilin	Bandell et al., 2007
		Iodoacetamide	Macpherson et al., 2007a
		Methyl isocyanate	Bessac et al., 2009
		Methyl p-hydroxybenzoate	Fujita et al., 2007
		Naphthalene metabolites	Lanosa et al., 2010
		Nicotine	Talavera et al., 2009
		Nitric oxide	Miyamoto et al., 2009
		Nitro-oleic acid (antiinflammatory nitric oxide derivative)	Sculptoreanu et al., 2010
		4-Oxo-nonenal (oxidative insult)	Andersson et al., 2008; Taylor-Clark et al., 2008
		Ozone	Taylor-Clark and Undem, 2010
		Tear gas (morphanthridine analogs)	Bessac et al., 2009; Gijsen et al., 2010
		Δ^9^-Tetrahydrocannabinol	Jordt et al., 2004
		Toluene diisocyanate	Taylor-Clark et al., 2009
		*Drugs*	
		N-Acetyl-p-benzo-quinoneimine (metabolite of acetaminophen)	Nassini et al., 2011
		Clioquinol	Andersson et al., 2009
		Chlordantoin	Maher et al., 2008
		Clotrimazole^d^	Meseguer et al., 2008
		Diclofenac	Hu et al., 2010
		1,4-Dihydropyridines (nicardipine, nifedipine, nimodipine)	Fajardo et al., 2008
		Disulfiram	Maher et al., 2008
		Flufenamic acid^e^	Hu et al., 2010
		Flurbiprofen	Hu et al., 2010
		Indomethacin	Hu et al., 2010
		Isoflurane	Eilers et al., 2010
		Ketoprofen	Hu et al., 2010
		Mefenamic acid	Hu et al., 2010
		Niflumic acid	Hu et al., 2010
		Propofol	Lee et al., 2008; Fischer et al., 2010

a Camphor activates TRPV1 and TRPV3 but inhibits TRPA1 (Xu et al., 2005).

b Menthol activates TRPM8 but inhibits TRPA1 (Macpherson et al., 2006).

c Cinnamaldehyde activates TRPA1 but inhibits TRPM8 (Macpherson et al., 2006).

d Clotrimazole activates both TRPA1 and TRPV1 but blocks TRPM8 (Meseguer et al., 2008).

e Flufenamic acid activates TRPA1 but inhibits TRPV1, TRPV3 and TRPM8 (Hu et al., 2010). For further details see Geppetti and Trevisani (2004), Vriens et al. (2008, 2009), Baraldi et al. (2010), and Boesmans et al. (2011).

**Table 2:** Select implications of TRPV1 in the physiology and pathophysiology of the digestive tract.

Tissue	Physiological or pathophysiological process	Type of evidence	References
Submandibular salivary gland	Acinar cell activation and salivary secretion in response to TRPV1 stimulation in humans	Induction by TRPV1 agonism	Ding et al., 2010; Zhang et al., 2010
Esophagus, stomach and \intestine	Stimulation of vagal and spinal afferent nerve fibers by capsaicin, acid and/or distension in rodents	Induction by TRPV1 agonism and prevention by TRPV1 knockout and/or antagonism	Maubach & Grundy, 1999; Su et al., 1999; Blackshaw et al., 2000; Rong et al., 2004; Sugiura et al., 2007; Bielefeldt & Davis, 2008; Peles et al., 2009
Esophagus, stomach and intestine	Hyperemia, bicarbonate secretion and protection of the mucosa from chemical injury (acid, ethanol, nosteroidal antiinflammatory drugs) in rats and humans	Induction by TRPV1 agonism	Hamid et al., 1981; Szolcsányi & Barthó, 1981; Holzer & Lippe, 1988; Holzer et al., 1989; Yeoh et al., 1995; Holzer, 1998, 2002; Aihara et al., 2005; Akiba et al., 2006a, 2008; Massa et al., 2006
Esophagus, stomach and intestine	Patients with erosive gastro-esophageal reflux disease, non-erosive reflux disease, irritable bowel syndrome, inflammatory bowel disease, idiopathic rectal hypersensitivity and fecal urgency, and Hirschsprung's disease	Upregulation of TRPV1 in the mucosa, with a correlation to pain sensitivity in irritable bowel syndrome and in inflammatory bowel disease with irritable bowel syndrome-like symptoms	Facer et al., 2001; Yiangou et al., 2001; Chan et al., 2003; Matthews et al., 2004; Bhat & Bielefeldt, 2006; Akbar et al., 2008, 2010; Guarino et al., 2010; Shieh et al., 2010
Esophagus, stomach, intestine and pancreas	Acid-induced oesophagitis, gastric acid-evoked injury of the stomach, trinitrobenzene sulfonic acid-induced pancreatitis and colitis in rodents	Upregulation of TRPV1 in vagal and spinal afferent neurons	Schicho et al., 2004; Banerjee et al., 2007; Miranda et al., 2007; Xu et al., 2007
Esophagus, stomach and small intestine	Abdominal pain in human volunteers	Induction by intraluminal capsaicin	Schmidt et al., 2004; Hammer, 2006a, 2006b; Hammer & Vogelsang, 2007; Hammer et al., 2008; Führer & Hammer, 2009; Kindt et al., 2009; Chen et al., 2010; van Boxel et al., 2010
Stomach	Behavioral pain response to intragastric acid challenge in rats	Prevention by TRPV1 antagonism	Lamb et al., 2003
Stomach and upper small intestine	Functional dyspepsia	Hypersensitivity to the algesic effect of capsaicin	Hammer et al., 2008
Stomach and upper small intestine	Functional dyspepsia	Beneficial effect of 5 week ingestion of capsaicin capsules	Bortolotti et al., 2002
Duodenum	Pain sensitivity to capsaicin exposure and balloon distension in humans	Beneficial effect of 4 week ingestion of capsaicin capsules	Führer & Hammer, 2009
Jejunum	Cardiovascular pain response to noxious distension in rats	Attenuation by TRPV1 ablation^a^	Lembeck & Skofitsch, 1982
Ileum	Ileitis induced by *Clostridium difficile* toxin A in rodents	Attenuation by TRPV1 antagonism	McVey & Vigna, 2001
Ileum and colon	Abdominal pain and viscerosomatic reflexes in humans	Intraluminal administration of capsaicin to ileostomy and colostomy patients	Drewes et al., 2003; Arendt-Nielsen et al., 2008
Colon	Abdominal pain in rodents	Induction by intraluminal capsaicin	Laird et al., 2001; Mansikka et al., 2004; Christoph et al., 2006
Colon	Hypersensitivity to balloon distension in humans	Ingestion of capsaicin capsules	Gonlachanvit et al., 2007
Colon	Abdominal pain and burning in patients with diarrhea-predominant irritable bowel syndrome	Hypersensitivity to ingestion of chili capsules	Gonlachanvit et al., 2009
Colon	Hyperalgesia caused by repetitive colorectal distension in rats	Prevention by TRPV1 antagonism	Ravnefjord et al., 2009
Colon	Sensitization of murine afferent nerve fibers to heat, acid and capsaicin by 5-hydroxytryptamine	Attenuation by TRPV1 knockout	Sugiura et al., 2004
Colon	Hyperalgesia to intraluminal acid and colorectal distension in rodents with acute experimental colitis	Attenuation by TRPV1 ablation, knockout and antagonism	Plourde et al., 1997; Delafoy et al., 2003; Jones et al., 2005, 2007; Miranda et al., 2007
Colon	Acute stress-evoked hyperalgesia to colorectal distension in rats subjected to maternal separation as neonates	Prevention by TRPV1 antagonism	van den Wijngaard et al., 2009b
Colon	Water avoidance stress-induced upregulation of TRPV1 in rat DRG neurons and hypersensitivity to colorectal distension	Prevention by TRPV1 antagonism	Hong et al., 2009
Colon	Post-inflammatory chemical and mechanical hyperalgesia in rodents	Attenuation by TRPV1 knockout and antagonism	Eijkelkamp et al., 2007; Jones et al., 2007; Winston et al., 2007; Wiskur et al., 2010
Colon	Colitis induced by dextran sulfate or trinitrobenzene sulfonic acid in rodents	Attenuation by TRPV1 knockout and antagonism	Kihara et al., 2003; Kimball et al., 2004; Miranda et al., 2007; Szitter et al., 2010
Colon	Colitis induced by trinitrobenzene sulfonic acid in rats	Upregulation of TRPV1 in and sensitization of colon-specific pelvic DRG neurons	De Schepper et al., 2008
Colon	Colitis-induced inhibition of gastric emptying in rats	Prevention by TRPV1 antagonism	De Schepper et al., 2008
Colon	Colitis induced by T-cell transfer in mice	Prevention by TRPV1 ablation	Gad et al., 2009
Colon	Secretory responses induced by distension or H_2_S in guinea-pig and human preparations	Attenuation by TRPV1 ablation and antagonism	Weber et al., 2001; Krueger et al., 2010
Anus	Intractable idiopathic pruritus ani	Attenuation by TRPV1 ablation	Lysy et al., 2003
Pancreas	Chronic pancreatitis induced by trinitrobenzene sulfonic acid in rats	Upregulation and sensitization of TRPV1 in pancreas-specific DRG neurons	Xu et al., 2007
Pancreas	Pancreatitis induced by caerulein in mice	Attenuation by TRPV1 antagonism	Nathan et al., 2001
Pancreas	Acid-evoked injury in a rat model of post-endoscopic cholangiopancreatography pancreatitis	Attenuation by TRPV1 ablation	Noble et al., 2008
Pancreas	Islet inflammation in non-obese diabetic mice (genetic model of type I diabetes)	Prevention by TRPV1 ablation	Razavi et al., 2006; Suri & Szallasi, 2008
Pancreas	Pain behavior, referred allodynia/hyperalgesia and spinal c-Fos expression associated with experimental pancreatitis in rodents	Attenuation by TRPV1 antagonism	Wick et al., 2006; Xu et al., 2007; Nishimura et al., 2010
Peritoneal cavity	Behavioral pain response to intraperitoneal injection of acetic acid or oleoylethanolamide in rodents	Attenuation by TRPV1 antagonism	Urban et al., 2000; Ikeda et al., 2001; Rigoni et al., 2003; Wang et al., 2005; Tang et al., 2007

a TRPV1 ablation refers to pretreatment with capsaicin or resiniferatoxin to defunctionalize afferent neurons.
